# Effective enhancement of the immunomodulatory capacity of canine adipose-derived mesenchymal stromal cells on colitis by priming with colon tissue from mice with colitis

**DOI:** 10.3389/fvets.2024.1437648

**Published:** 2024-08-08

**Authors:** Yuyo Yasumura, Takahiro Teshima, Tomokazu Nagashima, Masaki Michishita, Yoshiaki Taira, Ryohei Suzuki, Hirotaka Matsumoto

**Affiliations:** ^1^Laboratory of Veterinary Internal Medicine, School of Veterinary Medicine, Faculty of Veterinary Science, Nippon Veterinary and Life Science University, Musashino, Japan; ^2^Research Center for Animal Life Science, Nippon Veterinary and Life Science University, Musashino, Japan; ^3^Laboratory of Veterinary Pathology, School of Veterinary Medicine, Faculty of Veterinary Science, Nippon Veterinary and Life Science University, Musashino, Japan

**Keywords:** mesenchymal stromal cell, priming, immunomodulatory, inflammatory bowel disease, chronic inflammatory enteropathy, dextran sulfate sodium-induced colitis, microenvironment, stem cell therapy

## Abstract

**Introduction:**

The therapeutic efficacy of mesenchymal stromal cells (MSCs) in inflammatory bowel disease is not completely known and is not consistent. Priming with inflammatory cytokines has been proposed to adapt MSCs to an inflammatory environment to have them ready to counteract it, but may have undesirable effects on MSCs, such as increased immunogenicity. In this study, we hypothesized that priming MSCs with inflamed intestinal tissue would more effectively enhance their therapeutic effect on intestinal inflammation.

**Methods:**

The capacity of canine adipose-derived MSCs (cADSCs) primed with colon tissue homogenates from mice with experimentally induced colitis or a combination of tumor necrosis factor-α and interferon-γ to inhibit T-cell proliferation was analyzed, along with their own apoptosis, proliferation, cell surface marker expression, and transcriptome. In addition, colitis mice were treated with the primed cADSCs to assess colitis severity and immune cell profile.

**Results:**

Priming with cytokines induced apoptosis, decreased cell proliferation, and major histocompatibility complex-II gene expression in cADSCs, but these adverse effects were mild or absent with colitis-tissue priming. cADSCs primed with colitis tissue reduced the severity of colitis via the induction of M2 macrophages and T-regulatory cells and suppression of T-helper (Th)1/Th17-cell responses, and their effects were comparable to those of cytokine-primed cells.

**Discussion:**

Our results emphasize the importance of the activation of MSCs by the appropriate microenvironment to maximize their therapeutic effect.

## 1 Introduction

Inflammatory bowel disease (IBD), including Crohn's disease and ulcerative colitis, is a naturally occurring gastrointestinal disorder in humans caused by multiple etiologies, such as genetic factors, environmental factors, and abnormalities in gut microbiota and immune response (1). Patients' quality of life is severely compromised by chronic gastrointestinal symptoms that repeatedly go into remission and recur, and by complications such as fistulas, abscesses, and colorectal cancer (2). Current treatments for human IBD include aminosalicylic acid, glucocorticoids, immunosuppressants, and tumor necrosis factor (TNF)-α inhibitors, but nearly 30% of patients do not respond to these treatments and 50% of patients become refractory over time (1, 2). Chronic inflammatory enteropathy (CIE) is a multifactorial gastrointestinal disorder that occurs spontaneously in dogs, and its symptoms, pathogenesis, and treatment are similar in many ways to those of human IBD (3). CIE is clinically classified by treatment responsiveness into food-responsive enteropathy, antimicrobial-responsive enteropathy, and immunosuppressant-responsive enteropathy (3). Dogs that do not respond to these treatments are classified as having non-responsive enteropathy and reportedly account for 5% to 27% of dogs with CIE (4). Against this background, the development of new therapeutic strategies for human IBD and canine CIE is desirable.

Since their discovery by Friedenstein in 1966, mesenchymal stromal cells (MSCs) have been intensively studied as a cell source for tissue regeneration because of their stemness, including self-renewal and multilineage differentiation potential (5). Recently, MSCs have attracted attention for their stromal properties, including immunomodulatory and angiogenic capacities mediated by secreted factors, and many studies on them have been conducted not only in human medicine but also in veterinary medicine as a cell source for treating inflammatory and immune-mediated diseases (5, 6). MSCs can be isolated from almost all tissues of the body, including bone marrow, adipose tissue, umbilical cord, dental pulp, muscle, and synovium. Adipose-derived mesenchymal stromal cells (ADSCs) are one of the most common cell sources in humans and dogs due to their relatively low invasiveness, high-yield isolation, and high proliferative potential (5, 6). In fact, therapeutic effects of ADSCs have been observed in dogs as well as in humans for cutaneous, musculoskeletal, ocular, hepatic, respiratory, and neurological diseases (6, 7).

MSCs are expected to be a novel treatment for IBD and CIE and, indeed, have been shown to effectively ameliorate gastrointestinal inflammation in a number of preclinical studies using experimental animal models (8). However, the success of these preclinical studies has not been replicated in clinical trials (9). This may be attributable to the heterogeneity of MSCs caused by differences in sources such as donors and tissues, autologous or allogeneic origin, manufacturing steps such as isolation, culture, and cryopreservation methods, and application steps such as administration methods and patient conditions (10, 11). In veterinary medicine, two studies have reported fairly promising therapeutic effects of canine ADSCs (cADSCs) on CIE, but both were open-label studies without control groups in a small number of dogs by the same research group (12, 13). Therefore, there is a need to conduct larger-scale clinical trials to verify the efficacy and safety of MSCs, as well as for efforts to stabilize the quality of MSC products.

Priming approaches have been proposed as a way to enhance the inconsistent therapeutic effects of MSCs (11, 14). Priming, also known as preconditioning or licensing, has been demonstrated in several studies to affect MSC function by modulating biological, biochemical, and biophysical factors to enhance therapeutic efficacy (14). Among these, priming MSCs with inflammatory cytokines or hypoxic conditions is the most studied approach to enhance their immunomodulatory capacity by mimicking the inflamed microenvironment in the body (15). However, priming with inflammatory cytokines such as TNF-α and interferon (IFN)-γ may have adverse effects on MSCs for therapeutic application, such as by increasing their immunogenicity (15). MSCs exist as pericytes or fibroblast-like cells in the stromal tissue of almost all organs and work to maintain tissue homeostasis (16). Once tissue inflammation or damage occurs, MSCs are activated in response to the microenvironment and contribute to tissue repair through immunomodulation and matrix remodeling (17, 18). Although the cues in the disease microenvironment that activate MSCs are still unclear, it is suggested that a variety of factors act in combination to enhance their repair capacity. However, to the best of our knowledge, no studies have examined whether canine MSCs enhance their therapeutic potential in response to the disease microenvironment.

Based on the above, the purpose of our study was to investigate whether priming canine MSCs with disease tissue including components of the disease microenvironment would enhance their therapeutic efficacy on the original disease. In the context of studies to improve the therapeutic efficacy of MSCs for CIE, canine MSCs primed with inflammatory intestinal tissue from dogs with enteritis should be administered to canine models of enteritis to determine if the therapeutic efficacy is enhanced, but such a canine model has not been established. Therefore, we decided to test our hypothesis using a mouse model of dextran sulfate sodium (DSS)-induced colitis, a preclinical model of IBD and CIE commonly used in MSC xenotransplantation studies (8). If this study demonstrates that priming with colitis tissue from colitis mice enhances the immunomodulatory and therapeutic efficacy of cADSCs on mice with DSS-induced colitis, it will provide the basis for further studies to evaluate the functional changes of canine MSCs primed with inflamed intestinal mucosal samples from dogs with CIE and their efficacy on CIE patients.

## 2 Results

### 2.1 Priming with colon tissue homogenate from mice with colitis enhances the capacity of cADSCs to inhibit lymphocyte proliferation in a concentration-dependent manner

**Figure 1:**
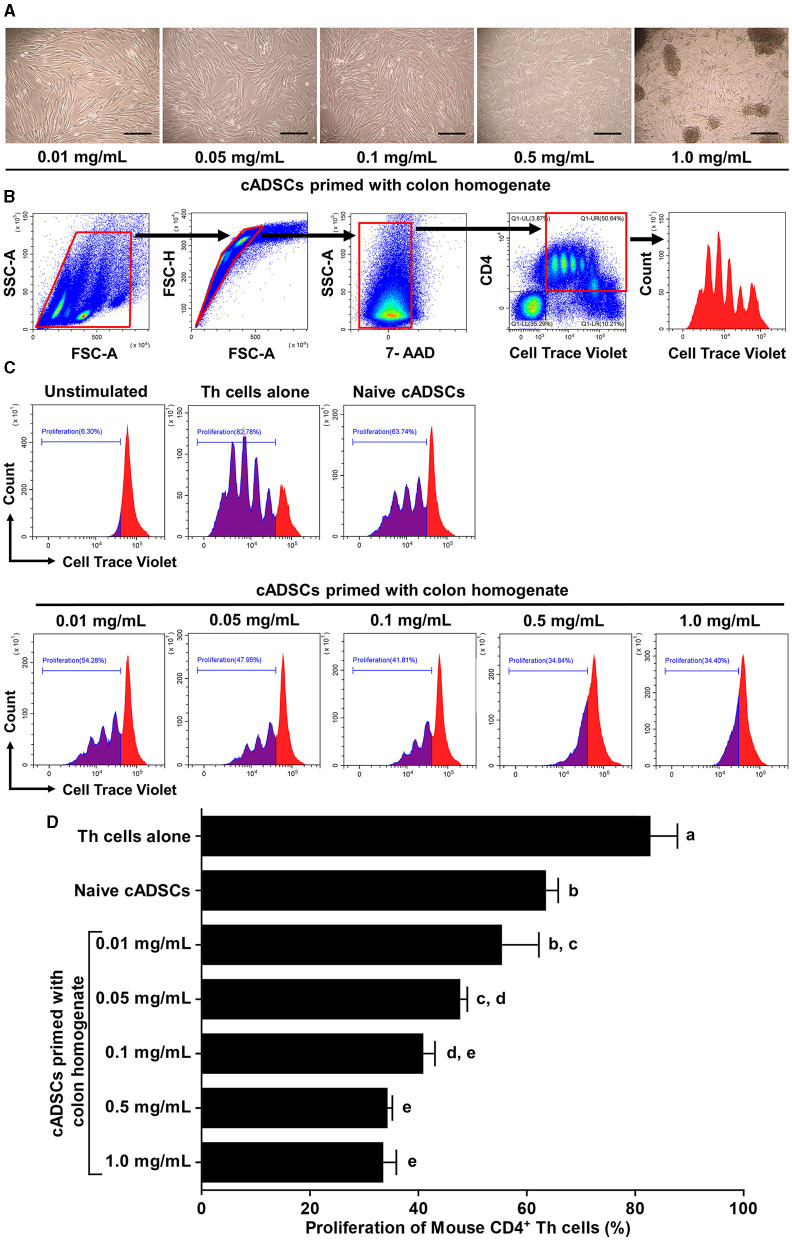
Effect of priming concentration of colon tissue homogenate from mice with colitis on the morphology of canine adipose-derived mesenchymal stromal cells (cADSCs) and their capacity to inhibit CD4^+^ T-helper (Th)-cell proliferation. **(A)** Microscopic morphology of cADSCs primed with colitis colon at graded concentrations. Scale bar = 200 μm. **(B)** Gating method in flow cytometry for analyzing Th-cell proliferation. Panels from left to right show gating of lymphocyte fractions, removal of doublets, removal of dead cells, gating of CD4^+^ cells, and histogram of cell trace violet-positive cells, with red boxes indicating gating regions. **(C)** Histogram of cell trace violet-positive cells showing CD4^+^ Th-cell division in each condition. **(D)** Comparison of CD4^+^ Th-cell proliferation in each condition. The proliferation of Th cells was quantified using the percentage of cell trace violet^low^ cells. The same lower-case letter beside the bars indicates that there is no significant difference between the groups (*p* > 0.05). Data are expressed as the mean ± standard deviation from experiments performed in triplicate; *n* = 5.

We first determined the concentration of colon tissue homogenate derived from colitis mice suitable for priming cADSCs. For this purpose, cADSCs were incubated with colon homogenates at graded concentrations for 24 h, followed by evaluation of primed cell morphology and cell viability. Colon priming at concentrations up to 0.1 mg/mL had no apparent effect on the morphology of the cADSCs, but at higher concentrations the cells were observed to “aggregate” (Figure 1A). The viability of primed cells, assessed by trypan blue exclusion, maintained above 98% at concentrations up to 0.5 mg/mL, but reduced mildly to about 94% at concentrations of 1.0 mg/mL (Supplementary Figure 1). After this experiment, primed cADSCs were co-cultured for 3 days with CD4^+^ T-helper (Th) cells derived from the spleen of colitis mice stimulated with anti-CD3/CD28-coated beads, followed by flow cytometry measurement of Th cell proliferation (Figure 1B). Priming with colon homogenate at concentrations up to 0.5 mg/mL enhanced the capacity of cADSCs to inhibit Th-cell proliferation in a concentration-dependent manner (Figure 1C). However, priming with colon homogenate at 1.0 mg/mL as the total protein concentration did not further enhance the capacity of cADSCs to inhibit Th-cell proliferation (Figure 1D). Therefore, the concentration of 0.1 mg/mL in colon priming that best enhanced the capacity of cADSCs to inhibit lymphocyte proliferation without affecting their cell morphology and survival was used in subsequent *in vivo* experiments.

### 2.2 cADSCs primed with colon homogenate strongly inhibit proliferation of CD4^+^ Th cells from mice with colitis, as do those primed with TNF-α+IFN-γ

**Figure 2:**
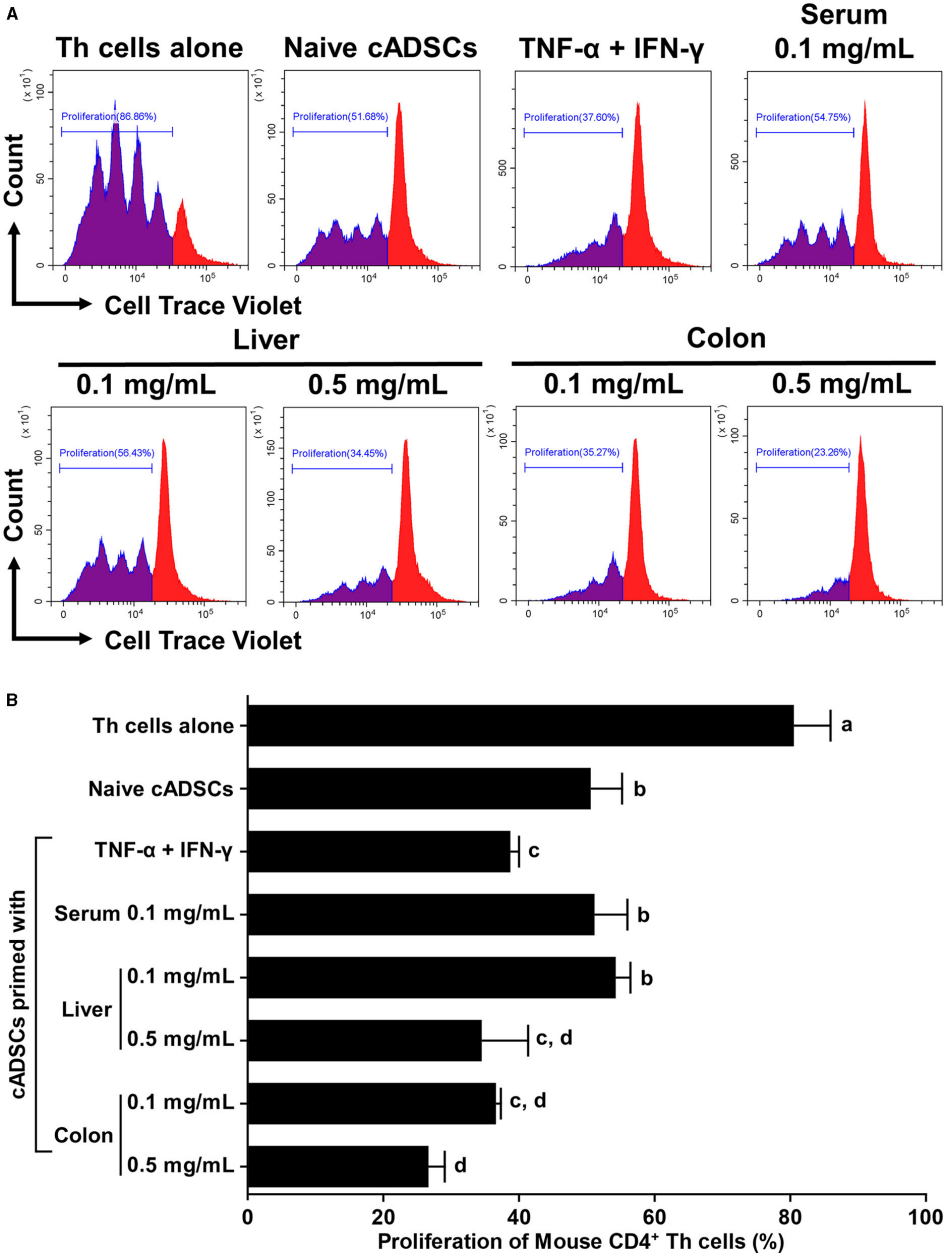
Effect of priming with different conditions on the capacity of cADSCs to inhibit Th-cell proliferation. **(A)** Histogram of cell trace violet-positive cells showing division of CD4^+^ Th cells co-cultured with primed cADSCs with each condition. **(B)** Comparison of Th-cell proliferation in each condition. cADSCs primed with colon homogenate at a low concentration inhibited Th-cell proliferation to the same extent as cADSCs primed with liver homogenate at a high concentration or tumor necrosis factor (TNF)-α+interferon (IFN)-γ. The same lower-case letter beside bars indicates that there is no significant difference between the groups (*p* > 0.05). Data are expressed as the mean ± standard deviation from experiments performed in triplicate; *n* = 5.

To compare the effects of priming on the capacity of cADSCs to inhibit Th-cell proliferation, cADSCs were primed with colon homogenate as well as purified inflammatory cytokines (TNF-α+IFN-γ), serum or liver homogenate from colitis mice for 24 h, after which co-culture assays were performed with primed cells and Th cells derived from mice with colitis. Based on the finding that colon priming enhances the capacity of cADSCs to inhibit Th-cell proliferation in a concentration-dependent manner up to 0.5 mg/mL, in this experiment, cADSCs were primed with mouse-derived substances at two different concentrations. However, priming with 0.5 mg/mL serum resulted in most cells aggregating after 24 h, so they were excluded from the experiment. Th-cell proliferation was significantly inhibited after 72 h in co-cultures with naïve or any primed cADSCs compared with that in mono-culture (Figure 2A). Priming with liver homogenate as well as colon homogenate significantly enhanced the capacity of cADSCs to inhibit Th-cell proliferation in a concentration-dependent manner (Figure 2B). The capacity to inhibit Th-cell proliferation was strongest for cADSCs primed colon homogenate at high concentrations, followed by cADSCs primed with colon homogenate at low concentrations, liver homogenate at high concentrations, and TNF-α+IFN-γ.

### 2.3 Colon priming could avoid adverse effects on cADSCs

**Figure 3:**
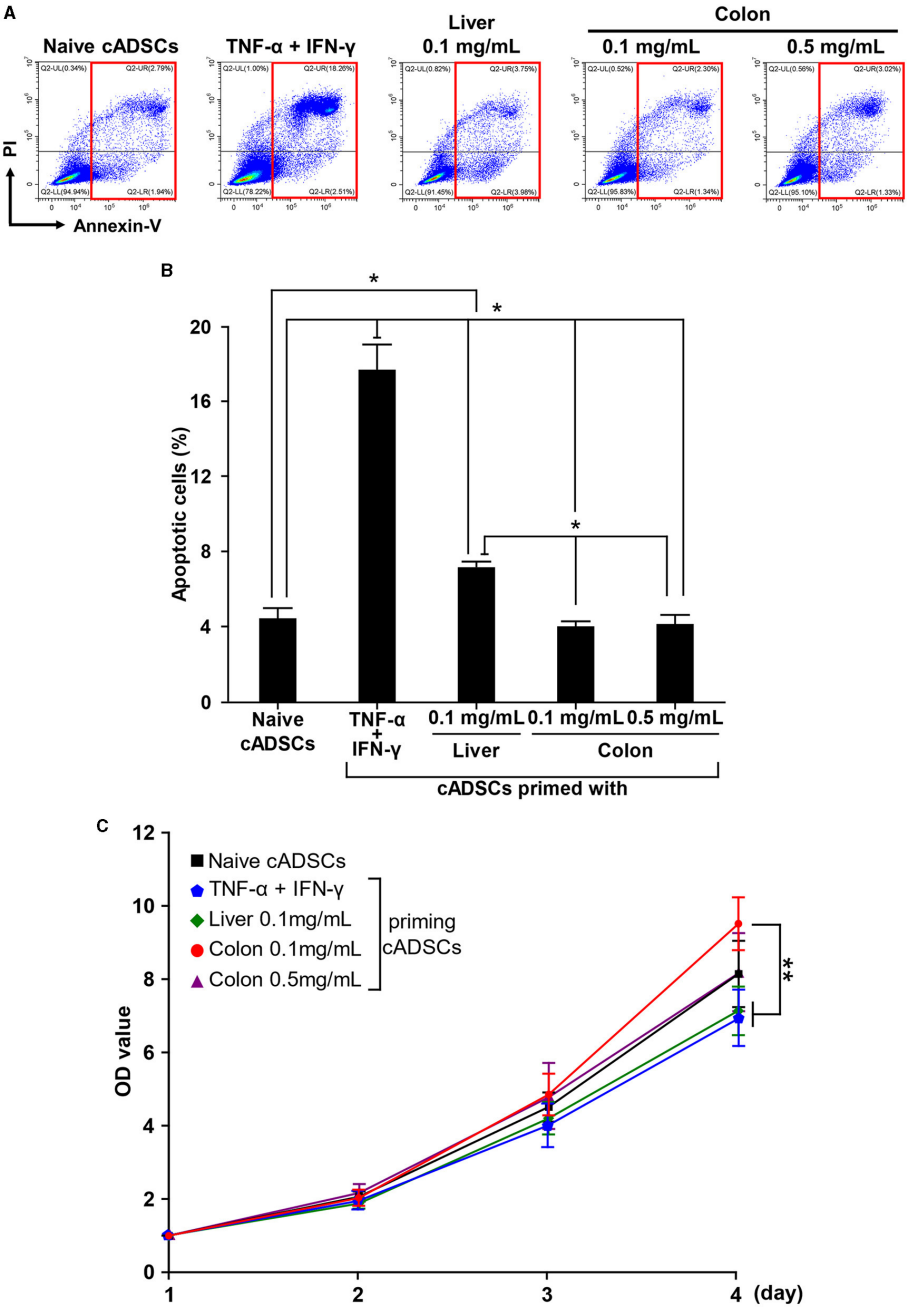
Different effects of various priming conditions on the properties of cADSCs. **(A)** Flow cytometric detection of apoptosis in primed cADSCs. The sum of the percentages of cells positive for Annexin V alone or in combination with propidium iodide (PI), indicated by red boxes, was measured as representative of apoptotic cells. **(B)** Comparison of the percentage of apoptotic cells in cADSCs primed with each condition. TNF-α+IFN-γ priming significantly induced apoptosis in cADSCs, but colon priming did not. **(C)** Proliferation of cADSCs after 1–4 days of priming measured spectrophotometrically. Optical density (OD) values after 2–4 days of priming were normalized by OD values after 1 day. Cell proliferation after 4 days of priming was best induced by priming with colitis colon at a low concentration. Data are expressed as the mean ± standard deviation from experiments performed in triplicate; *n* = 5; *p < 0.05, **p < 0.01.

Priming may affect the survival, proliferation, and immunogenicity of MSCs, as well as their immunomodulatory capacity (14). Therefore, we next investigated the effect of priming with diseased tissue on the properties of cADSCs. To examine the effect of priming on survival, cADSCs were primed with TNF-α+IFN-γ, liver homogenate, and a low or high concentration of colon homogenate for 24 h, after which the frequency of Annexin-V and propidium iodide (PI)-positive apoptotic cell was measured by flow cytometry (Figure 3A). Priming with these conditions was chosen because TNF-α+IFN-γ application is a well-studied priming method, liver represents a non-primary lesion organ in colitis, and low and high concentrations of colon homogenate allow the study of concentration-dependent effects. Apoptosis of cADSCs was induced drastically by TNF-α+IFN-γ priming and mildly by liver priming, while colon priming did not induce apoptosis at levels exceeding that of naive cADSCs, even at a high concentration (Figure 3B).

As with apoptosis, cADSCs were primed for 24 h under each condition, followed by passaging of primed cADSCs and analysis of cell proliferation after 1–4 days. Differences in the proliferation of cADSCs primed with different conditions were observed day by day, and after 4 days, cADSCs primed with colon homogenate at a concentration of 0.1 mg/mL showed better cell proliferation than those under the other conditions (Figure 3C). In contrast, the proliferation of cADSCs primed with TNF-α+IFN-γ or liver homogenate was rather suppressed compared with that of naive cADSCs.

### 2.4 cADSCs primed with colon homogenate were showed enriched expression of genes associated with cell proliferation in addition to inflammation-related genes

**Figure 4:**
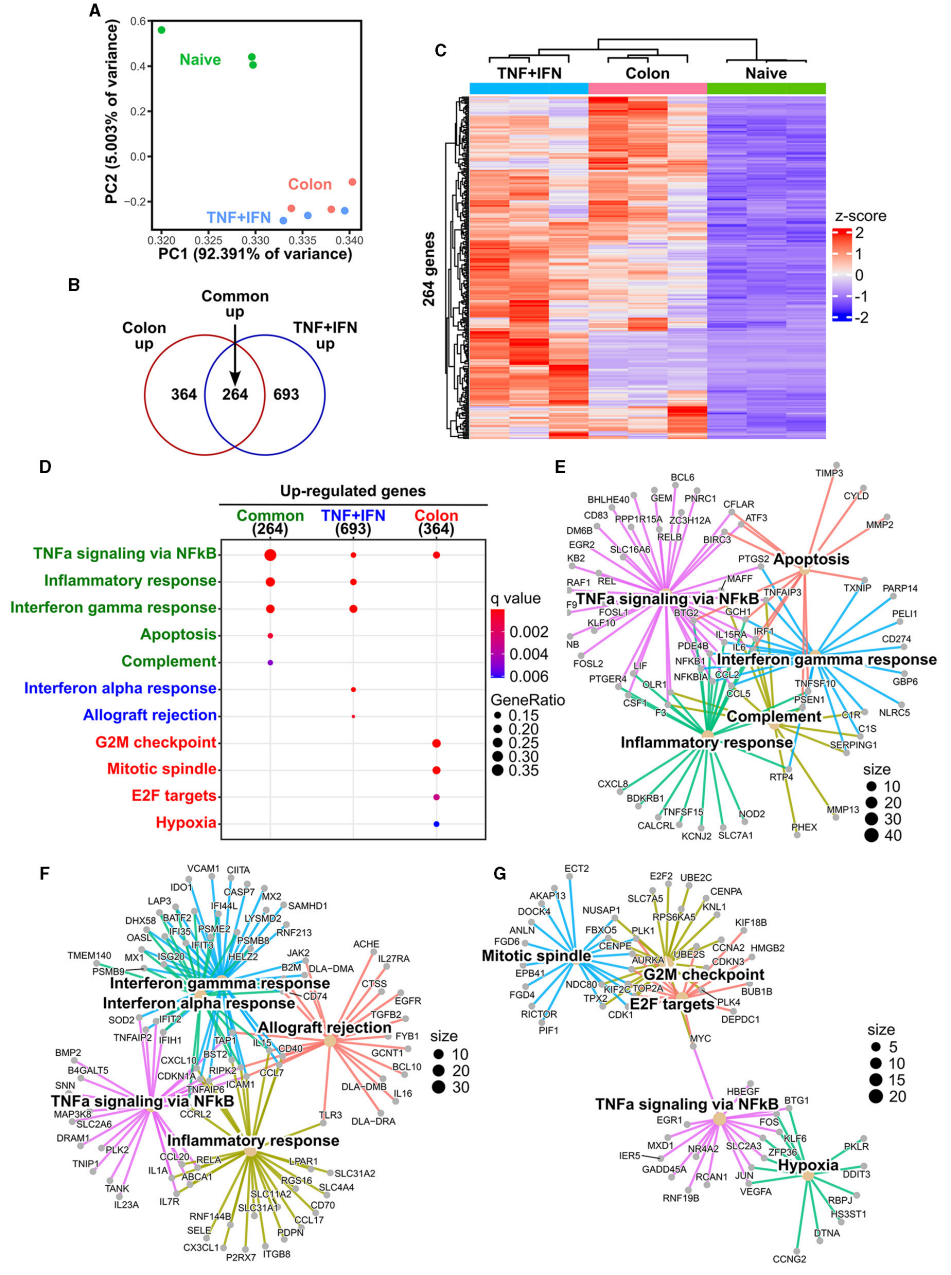
Effects of TNF-α+IFN-γ priming and colon priming on the transcriptome of cADSCs. **(A)** Biplot obtained from principal component analysis (PCA) for transcriptomes of naive cADSCs and those primed with TNF-α+IFN-γ or colon homogenate. **(B)** Venn diagram of genes significantly upregulated by priming with TNF-α+IFN-γ or colon homogenate. Differentially expressed gene (DEG) analysis was performed using DESeq2; *n* = 3; fold change >2, false discovery rate (FDR) < 0.05. **(C)** Integrated heatmap of genes commonly upregulated in both types of priming. **(D)** Top-ranked functional pathways enriched in gene sets upregulated by priming, including 264 common genes (green), 693 genes specific to TNF-α+IFN-γ priming (blue), and 364 genes specific to colon priming (red); FDR < 0.05. **(E)** Gene-concept network (cnet) plot of pathways enriched for upregulated gene sets common to each type of priming. **(F)** cnet plot of pathways enriched for gene sets specifically upregulated by TNF-α+IFN-γ priming. **(G)** cnet plot of pathways enriched for gene sets specifically upregulated by colon priming.

To further characterize the biological response of cADSCs to priming with colon homogenate representing diseased tissue, gene expression profiling was performed using RNA sequencing (RNA-seq) and the results were compared to the transcriptomes of naive and TNF-α+IFN-γ-primed cADSCs. Primary analysis of sequencing reads obtained by RNA-seq detected the expression of 17,863 genes among naive cADSCs, and cADSCs primed with TNF-α+IFN-γ or colon homogenate (Supplementary Table 1). Principal component analysis (PCA) showed that priming markedly altered the gene expression of cADSCs, with variation among the different strains of cADSCs (Figure 4A). In particular, to identify the genes of cADSCs affected by priming in common between the two different conditions and the genes specifically affected by each type of priming, differentially expressed genes (DEGs) were detected from each dataset using DESeq2 and Venn diagram analysis was performed. TNF-α+IFN-γ priming and colon priming upregulated 957 and 628 genes, respectively, of which 264 genes were in common (Figures 4B, C, Supplementary Figures 2A, B, Supplementary Table 2). At the same time, the downregulated genes numbered 371 and 489 for TNF-α+IFN-γ priming and colon priming, respectively, of which 186 genes were in common (Supplementary Figures 2A–D, Supplementary Table 3). Enrichment analysis based on the Molecular Signatures Database (MSigDB) hallmark gene set was performed to reveal the biological relevance of the gene sets that were specifically or commonly up- or downregulated by each priming condition. The top-ranked pathways associated with the 264 commonly upregulated genes were inflammation-related pathways such as TNF-α signaling via nuclear factor-kappa B (NFκB), inflammatory response, IFN-γ response, and complement, as well as apoptosis (Figures 4D, E, Supplementary Table 4). Similarly, the pathways associated with the 693 genes that were specifically upregulated by TNF-α+IFN-γ priming were inflammation-related pathways, including TNF-α signaling via NFκB, inflammatory response, IFN-γ response, and IFN-α response, but also allograft rejection pathways (Figure 4F). In contrast, pathways associated with the 364 genes specifically upregulated in colon priming were cell proliferation-related pathways such as the G2M checkpoint, mitotic spindle, and E2F targets, as well as hypoxia (Figure 4G). Enrichment analysis of the gene sets downregulated by the different priming conditions revealed that late estrogen response was associated with both types of priming; epithelial–mesenchymal transition was specifically associated with TNF-α+IFN-γ priming; and E2F targets, cholesterol homeostasis, and Notch signaling pathways were specifically associated with colon priming (Supplementary Figures 2E–H, Supplementary Tables 4, 5).

### 2.5 Colon priming improves the therapeutic effect of cADSCs on colitis to the same extent as TNF-α+IFN-γ priming

**Figure 5:**
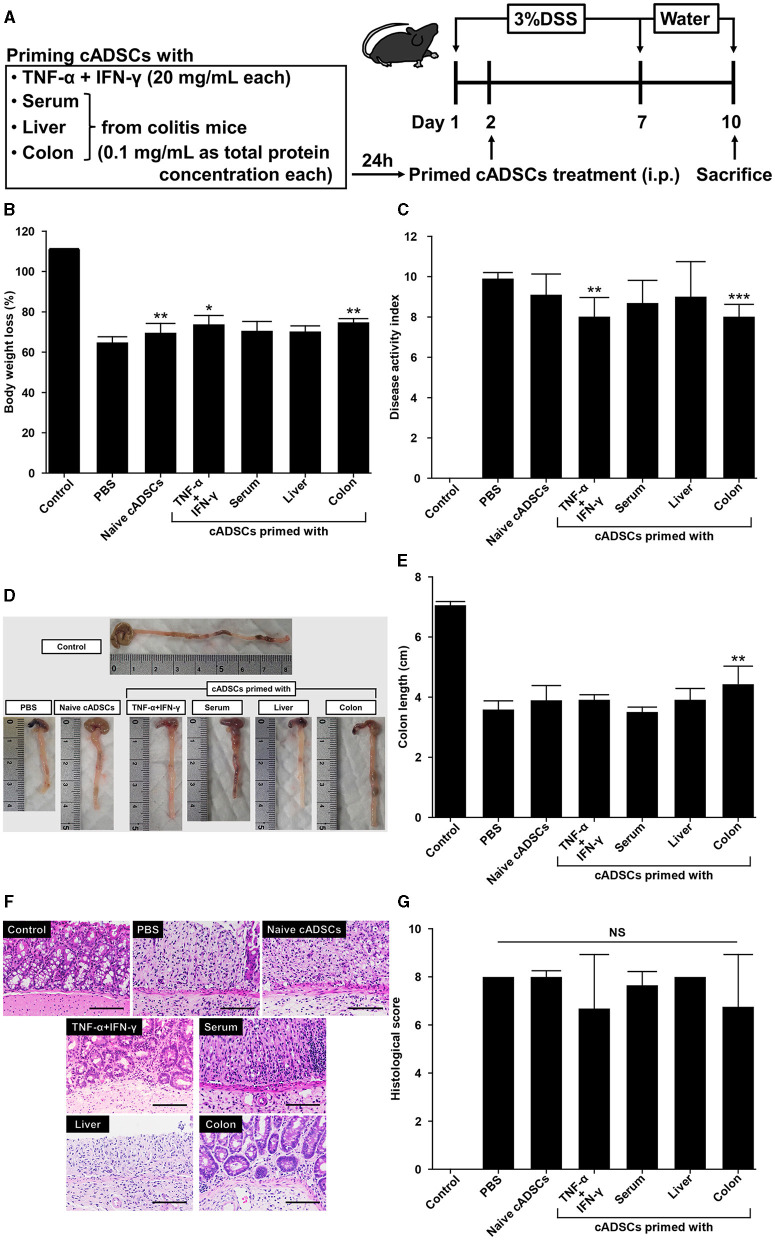
Effect of priming conditions on the therapeutic efficacy of cADSCs in dextran sulfate sodium (DSS)-induced colitis. **(A)** Scheme for priming and administering cADSCs and induction of colitis by DSS. **(B)** Comparison of body weight loss on day 10 in mice receiving cADSCs primed with each condition. Control represents healthy mice that received neither DSS nor cADSCs. **(C)** Comparison of disease activity index (DAI) at day 10 of the experiment. Colon-primed cADSCs most significantly reduced DAI. **(D)** Gross images of the colon of mice treated with cADSCs primed with each condition. The length of the colon, excluding the cecum, was measured. **(E)** Colon-primed cADSCs significantly improved colon shortening. **(F)** Histological images of the colon of mice treated with primed cADSCs. Some mice treated with cADSCs primed with TNF-α+IFN-γ or colon homogenate had relatively mild mucosal epithelial injury. Bar = 100 μm. **(G)** Comparison of histological scores in each group. Data are expressed as the mean ± standard deviation; *n* = 5; *p < 0.05, **p < 0.01, ***p < 0.001, NS, not significant.

To assess whether exposure to diseased tissues specifically enhances the therapeutic effect of cADSCs on the original disease, cADSCs primed with each condition were administered intraperitoneally to mice on day 2 of colitis induction by DSS (Figure 5A). The mice used in a series of experiments investigating the therapeutic effects of primed cADSCs *in vivo* were different from those used for homogenate preparation and co-culture assays. On day 10 of the experiment, compared with that in the colitis mice receiving phosphate-buffered saline (PBS), body weight loss was mildly but significantly suppressed in colitis mice receiving naive cADSCs and cADSCs primed with TNF-α+IFN-γ or colon homogenate (Figure 5B), and disease activity index (DAI) was significantly reduced in mice receiving cADSCs primed with TNF-α+IFN-γ or colon homogenate (Figure 5C). Meanwhile, in the assessment of the length of the mouse colon after sacrifice, only cADSCs primed with colon homogenate showed improvement in shortening (Figures 5D, E). Histological evaluation of the colon showed no significant ameliorative effect of primed cADSCs, although some mice treated with cADSCs primed with TNF-α+IFN-γ or colon homogenate had relatively mild histological severity (Figures 5F, G).

### 2.6 cADSCs primed with colon homogenate markedly shift macrophage polarity to M2 phenotype

**Figure 6:**
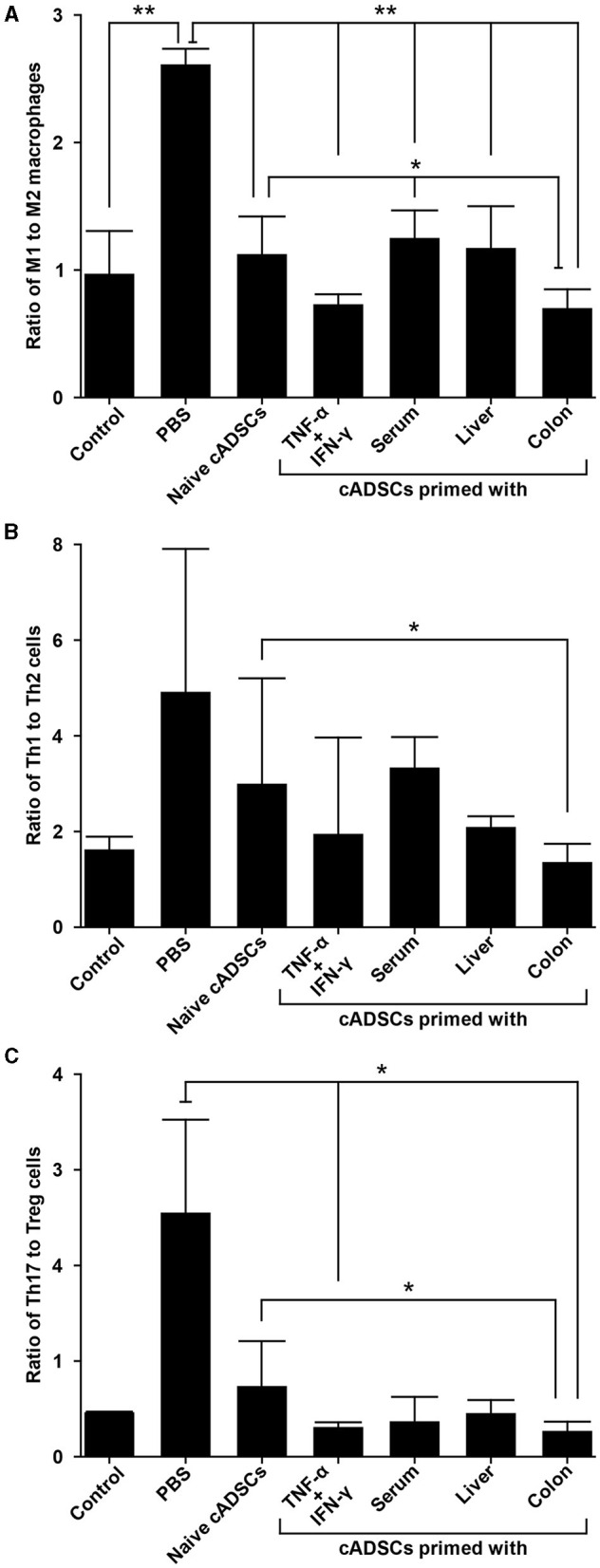
Differential capacity of cADSCs primed with variable conditions to modulate macrophage polarity and Th cell paradigm. **(A)** The ratio of M1 to M2 macrophage frequencies. cADSCs primed with TNF-α+IFN-γ or colon homogenate markedly shifted macrophage polarity toward M2 dominance. **(B)** Balance of the Th1/Th2-cell paradigm as indicated by the ratio of Th1 to Th2-cell frequencies. The cADSCs primed with colon homogenate modulated the Th1/Th2 balance by inhibiting Th1-cell activation. **(C)** Balance of the Th17/T regulatory (Treg)-cell paradigm as indicated by the ratio of Th17 to Treg cells. Priming with any condition effectively enhanced the capacity of cADSCs to suppress Th17-cell responses and induce Treg-cells, but the effect was particularly pronounced with TNF-α+IFN-γ or colon-priming. Data are expressed as the mean ± standard deviation; *n* = 5; *p < 0.05, **p < 0.01.

We next performed phenotypic analysis of macrophages to determine the modulatory effects of primed cADSCs on these cells, which are important mediators in the pathogenesis of DSS-induced colitis in mice (19). Peritoneal macrophages from colitis mice treated with cADSCs primed with each condition were isolated and the frequencies of F4/80^+^ CD80^+^ proinflammatory M1 macrophages and F4/80^+^ CD206^+^ anti-inflammatory M2 macrophages were determined using flow cytometry. Both naive cADSCs and cADSCs primed with any condition reduced F4/80^+^ CD80^+^ M1 macrophages, which were drastically increased by colitis induction, but cADSCs primed with TNF-α+IFN-γ or colon homogenate in particular significantly and markedly reduced them (Supplementary Figures 3A, B). Concurrently, F4/80^+^ CD206^+^ M2 macrophages increased to higher levels than in healthy control mice with the administration of any cADSCs, and were most increased with administration of cADSCs primed with colon homogenate (Supplementary Figures 3C, D). The ratio of M1 to M2 phenotypes of macrophages was greatly polarized toward M1 dominance by induction of colitis, but was significantly skewed toward M2 dominance by administration of cADSCs primed with any conditions, especially by administration of cADSCs primed with colon homogenate (Figure 6A).

### 2.7 cADSCs primed with colon homogenate effectively suppress Th1/Th17-cell responses

Among the various Th-cell subsets, activation of the Th1/Th17-cell response, characterized by a cytokine profile featuring components such as TNF-α, IFN-γ, interleukin (IL)-6, and IL-17, also contributes to the development of the acute phase of DSS-induced colitis (20). Therefore, we investigated the effect of priming with diseased tissue on the capacity of cADSCs to modulate an imbalanced Th-cell paradigm by the phenotypic analysis of CD4^+^ Th cells from mice with colitis. To this end, CD4^+^ Th cells were isolated from the spleens of colitis mice treated with cADSCs primed with each condition, and the frequencies of Th1 cells expressing IFN-γ as an intracellular cytokine, Th2 cells expressing IL-4, Th17 cells expressing IL-17A, and T-regulatory (Treg) cells expressing both CD25 on the cell surface and the transcription factor Foxp3 in the nucleus were determined using flow cytometry.

The frequency of CD4^+^ IFN-γ^+^ Th1 cells did not show statistically significant changes in any of the treated mice compared to the control group (Supplementary Figures 4A, B). Even though the frequency of Th1 cells tended to increase in colitis mice without cADSC treatment and not in mice with primed cADSC treatment, the reason statistical significance was not detected is suggested by the high variability in the colitis mice without cADSC. For CD4^+^ IL-4^+^ Th2 cells, the induction of colitis by DSS and treatment with any cADSCs did not cause consistent and obvious changes (Supplementary Figures 4C, D). The ratio of Th1 to Th2 cell frequencies, representing the Th1/Th2 balance, was significantly reduced in mice treated with cADSCs primed with colon homogenate to the same level as in healthy controls, in contrast to the level in mice treated with naive cADSCs (Figure 6B). Again, because of the high variability in the PBS group, no statistically significant difference between the PBS group and the colon primed group was detected.

The naive and all primed cADSCs consistently reduced the frequency of CD4^+^ IL-17A^+^ Th17 cells increased by colitis induction, similar to the suppression pattern in Th1 cells, and especially cADSCs primed with colon homogenate significantly reduced Th17 cells (Supplementary Figures 5A, B). In contrast, the frequency of CD4^+^ CD25^+^ Foxp3^+^ Treg cells was clearly increased in mice treated with all primed cADSCs, and those treated with cADSCs primed with TNF-α+IFN-γ or colon homogenate in particular showed significant and marked increases in this frequency compared with PBS-treated mice (Supplementary Figures 5C, D). The ratio of Th17- to Treg-cell frequencies as a measure of the balance of the Th17/Treg-cell paradigm was significantly reduced in mice treated with cADSCs primed with TNF-α+IFN-γ or colon homogenate compared with that in mice without cADSC treatment (Figure 6C). In summary, all priming methods enhanced the capacity of cADSCs to suppress Th1/Th17-cell responses and induce Treg cells in colitis mice, with colon priming having the strongest enhancing effect.

## 3 Discussion

Various priming approaches have been proposed to improve the therapeutic efficacy of MSCs, including cytokines, growth factors, hypoxia, drugs, and biomaterials (14). Among them, the priming of MSCs with inflammatory cytokines aimed at increasing the secretion of anti-inflammatory and immunomodulatory factors has been the subject of numerous studies. Yang et al. reported that priming cADSCs with a combination of TNF-α and IFN-γ markedly enhanced the capacity of these cells to suppress the expression of proinflammatory cytokines in lipopolysaccharides (LPS)-activated macrophage cell lines and canine peripheral blood mononuclear cells via upregulation of the cyclooxygenase (COX)-2/prostaglandin E2 (PGE2) pathway (21). The same group also showed that TNF-α-primed cADSCs ameliorated colitis by promoting the polarization of M2 macrophages in mice with DSS-induced colitis via the hypersecretion of PGE2 and TNF-α-stimulated gene/protein 6 (TSG-6) (22), and that extracellular vesicles (EVs) derived from cADSCs primed with TNF-α and IFN-γ effectively suppressed colitis by inducing Treg cells and M2 macrophages (23). Consistent with the above studies, priming of cADSCs with a combination of TNF-α and IFN-γ in this study drastically enhanced the capacity of cADSCs to induce M2 macrophages and Treg cells, suppress Th1/Th17-cell responses, and inhibit Th-cell proliferation, thereby reducing the severity of colitis, with the upregulation of COX-2 and TSG-6 (Supplementary Table 1). Enrichment analysis showed that pathways such as TNF-α signaling via NFκB, IFN-γ responses, and inflammatory responses were enriched as expected, but surprisingly these pathways were also commonly enriched upon priming with colitis tissue. Furthermore, the enhancing effect of cADSCs on immunomodulatory capacity was equally observed upon priming with colitis tissue. The colon of mice with DSS-induced colitis expresses high levels of TNF-α and IFN-γ compared with that of healthy mice, but at picogram levels (24, 25). Therefore, cytokine concentrations much lower than those used in this study (20 ng/mL) may be sufficient to enhance the immunomodulatory capacity of cADSCs, and the inclusion of cytokines at concentrations and types more closely resembling the inflammatory environment may appropriately enhance the therapeutic effect of cADSCs. This is supported by studies reporting that exposure of human ADSCs to synovial fluid from patients with rheumatoid arthritis or plasma from patients with graft-vs.-host disease, which contain picogram levels of multiple inflammatory cytokines such as TNF-α, exerts better immunomodulatory effects (26, 27).

Priming is a good way to enhance MSC function, but one must consider that it may affect MSC survival, proliferation, and immunogenicity (15). In this regard, Li et al. showed that treatment of mouse bone marrow-derived MSCs (BMSCs) for 48 h with 10 ng/mL each of TNF-α and IFN-γ synergistically induced cell apoptosis, which was mediated by nitric oxide (28). In addition, Domenis et al. reported that priming of human ADSCs with graded concentrations of TNF-α and IFN-γ from 10 to 40 ng/mL for 48 h caused concentration-dependent changes in cell morphology and decreased proliferation (29). Regarding the effect on immunogenicity, Montesinos et al. demonstrated that stimulation of human BMSCs with a low concentration of TNF-α (0.5 ng/mL) or IFN-γ (5 ng/mL) for 24 h increased the expression of major histocompatibility complex (MHC)-I (30). Meanwhile, MHC-II expression of human BMSCs was not induced by TNF-α, but was markedly induced by IFN-γ, after 24 h (31, 32). Similar to these reports, in the present study, cADSC priming with the combination of TNF-α and IFN-γ induced apoptosis, decreased cell proliferation, and MHC-II gene (*DLA-DRA* and *DLA-DQA1*) expression, while enrichment analysis revealed enrichment of the apoptotic and allograft rejection pathways. A marked upregulation of MHC-II gene expression in cytokine-primed cells was revealed by RNA-seq (Supplementary Table 1). In contrast, priming of cADSCs with colitis tissue did not induce cell apoptosis, even at high concentrations, but rather promoted cell proliferation, and MHC-II gene expression was marginal. Priming with colitis tissue also showed upregulation of the apoptotic pathway in common, while the upregulation of cell proliferation and hypoxic pathways was specifically observed. Several studies have shown that priming MSCs under hypoxic conditions improves the survival and proliferative potential of the cells (33, 34). The detailed mechanisms by which the hypoxia and cell proliferation pathways were upregulated are unknown, but activation of these pathways may have protected cADSCs primed with colitis tissue from apoptosis. With regard to immunogenicity, priming human BMSCs with IL-17 alone or a cocktail of IL-1β, IL-6, and IL-23, cytokines secreted by Th17 cells, has been reported to enhance immunomodulatory capacity without increasing the expression of MHC-II or co-stimulatory molecules (35, 36). Considering the activation of Th17 cells that occurs in the colon of mice with DSS-induced colitis, it is suggested that not only low concentrations of TNF-α and IFN-γ in colitis tissue, but also Th17 cytokines, may be responsible for the enhanced function of cADSCs without increasing their immunogenicity. Similar priming effects that enhance function without adversely affecting MSCs are also provided by exposure of human ADSCs to the inflammatory environment produced by activated T cells, synovial fluid from rheumatoid arthritis patients, or plasma from graft-vs.-host disease patients (26, 27, 37).

MSCs are well known to change their properties and functions in response to the surrounding microenvironment (18). Therefore, the aim of this study was to investigate whether *ex vivo* exposure of cADSCs to inflamed colon tissue from mice would more effectively enhance the therapeutic effect of cADSCs on colitis in mice. DSS-induced colitis is a representative disease model used in preclinical studies of human IBD and canine CIE (38). Actually, some studies have reported that xenotransplantation of canine MSC ameliorate DSS-induced colitis in mice via immunomodulatory effects (21, 22, 39). In this study, to determine whether the original disease microenvironment enhances the immunomodulatory and therapeutic effects of canine MSCs on DSS-induced colitis in mice, cADSCs were primed with colon homogenates from colitis mice rather than dogs and the enhancing effects were compared to other priming conditions. DSS is toxic to mucosal epithelial cells of the colon, and when 40–50 kDa DSS is added at a concentration of 1–5% to drinking water and fed to mice for 5–10 days, it disrupts the integrity of the epithelial barrier and causes acute colitis (40). In addition to epithelial cell damage, macrophages play an important role in the pathogenesis of colitis, and stimulated and subsequently activated proinflammatory M1 macrophages not only cause direct colonic mucosal injury via the secretion of proinflammatory cytokines such as TNF-α, IL-1β, and IL-6, but also cause the infiltration of neutrophils and CD4^+^ Th cells into the mucosal epithelium via the secretion of IL-23 (41). Meanwhile, the adaptive immune system is not essential for the development of colitis, but the Th1/Th17-cell response, characterized by cytokines such as IFN-γ, IL-6, and IL-17, contributes to the progression of colitis (20, 42). Although the mechanism by which MSCs improve the pathogenesis of this model is not fully understood, they have been reported to improve the severity of colitis by reprogramming macrophages, modifying the Th-cell paradigm, and inducing regulatory immune cells via the secretion of indoleamine 2,3-dioxygenase (IDO), PGE2, transforming growth factor-β1, TSG-6, and EVs, among others (8). It has also been reported that intravenously administered rat BMSCs engraft onto damaged colon epithelium and restore the integrity of the epithelial barrier by rebuilding the tight junction protein claudin (43). Consistent with many previous reports, our intraperitoneally administered cADSCs, even naive ones, shifted the macrophage phenotype from M1 to M2 polarity, suppressed activation of Th1/Th17-cell responses, and directly inhibited Th-cell proliferation. However, their immunomodulatory capacity and were particularly enhanced in cADSCs primed with a combination of the inflammatory cytokines TNF-α and IFN-γ and in cADSCs primed with colon homogenate from colitis mice. These results emphasize the importance of activating MSCs by exposure to an inflammatory environment in order to maximize their therapeutic efficacy.

Although MSCs are not constitutively immunosuppressive, several studies have shown that their immunomodulatory function is activated by the host inflammatory environment (44). Therefore, in DSS-induced colitis, delivery of MSCs to inflamed colonic tissues may effectively activate their immunomodulatory function. Differences in the migration of MSCs to the inflamed colon that depend on the route of administration have been reported. For example, Castelo-Branco et al. showed that the intraperitoneal administration of allogeneic ADSCs to rats with trinitrobenzene sulfonic acid (TNBS)-induced colitis improved inflammation via the migration of ADSCs into the inflamed colon, but intravenous administration did not improve inflammation as the cells did not migrate into the colon (45). Li et al. also reported that the intravenous administration of human BMSCs to mice with TNBS-induced colitis resulted in most of them being trapped in the lungs and spleen, but the overexpression of CXCL2 on the cells markedly increased migration to the colon (46). In contrast, Gonçalves et al. reported that allogeneic ADSCs administered intravenously to mice with DSS-induced colitis induced high levels of IFN-γ in mouse serum, thereby activating the immunomodulatory function of ADSCs and showing increased T-cell apoptosis in the inflamed colon and improvement of inflammation, although this did not occur upon intraperitoneal administration (47). Furthermore, Pan et al. showed that allogeneic umbilical cord-derived MSCs administered intraperitoneally or intravenously to mice with DSS-induced colitis migrate into the colon by either route and improve colitis by improving local microcirculation, repairing epithelial barriers, and exerting immunomodulatory effects, with the effect of intraperitoneal administration being shown to be superior (48). Although the biodistribution of administered cADSCs was not analyzed in this study, it is possible that some of the intraperitoneally administered cADSCs migrated to the inflamed colon and were activated by its microenvironment, as suggested by the fact that even naive cADSCs exhibited immunomodulatory effects. Thus, intraperitoneal administration may be a more appropriate route of administration to enhance the therapeutic effect of MSCs in colitis in mice. However, the intraperitoneal administration of MSCs is not practical in large animals such as dogs or in humans due to the large volume of their abdominal cavity. With the exception of fistula-type Crohn's disease, local administration of MSCs is difficult in canine CIE and human IBD because of the diffuse or multiple lesions in the intestine, which is much larger and longer than in mice, and MSCs are almost always administered intravenously (8, 10). Although no studies have yet examined the biodistribution of intravenously administered MSCs in dogs with CIE or patients with IBD, intravenously administered MSCs in these species are often trapped early in the lungs and then redistributed to the spleen, liver, and kidney, but rarely in the intestinal tract (49). Furthermore, proteins, including cytokines, in the blood do not accurately reflect the state of inflammation in the gastrointestinal tract, as evidenced by the absence of established blood biomarkers (50). In summary, because intravenously administered MSCs in dogs and humans are less exposed to the inflammatory microenvironment, priming MSCs with inflammatory tissue may be a promising approach for successful MSC therapy not only in rodent experimental animal models but also in canine CIE and human IBD patients.

Hepatobiliary disorders are common extraintestinal manifestations of IBD (51). Similarly, in DSS-induced colitis, disruption of the colonic mucosal epithelial barrier increases intestinal permeability, allowing inflammatory cytokines and toxins such as LPS to reach the liver via the portal vein and cause inflammatory damage (52). In addition, during the acute phase of colitis caused by DSS, serum from C57BL/6J mice exhibits a cytokine expression pattern characterized by TNF-α, IFN-γ, and IL-6 from M1 macrophages/Th1 cells and IL-17 from Th17 cells similar to the expression pattern found in colon (24, 25). Therefore, we also primed cADSCs with serum and liver as non-colon tissues derived from colitis mice to test the specificity of priming with colitis tissue in enhancing the therapeutic effect of MSCs on colitis. Our results showed that priming with colitis tissue enhanced the immunomodulatory and therapeutic effects of cADSCs more than priming with other tissues, suggesting that the enhancing effects were specific. Given that the factors contained in each tissue sample are diverse, including not only cytokines but also EVs, miRNAs, and extracellular matrix, this study cannot clarify the mechanisms involved in the enhancement of cADSC function by priming, but it is possible that not only factors induced by inflammation but also components of colon tissue played an important role. To further validate this site-specific potentiation effect, it is necessary to compare the therapeutic effect of cADSCs primed with liver homogenates or colon homogenates on hepatitis models, for example.

Taken together, priming with colitis tissue homogenate enhanced the immunomodulatory capacity of cADSCs as much as priming with a combination of TNF-α and IFN-γ, a priming method that is well known to enhance the immunosuppressive capacity of MSCs. Furthermore, priming with colitis tissue enhanced the function of cADSCs without inducing apoptosis, decreased cell proliferation, or immunogenicity. Therefore, priming MSCs with diseased tissues may be an alternative method of priming MSCs to that of cytokines. We believe that if future studies demonstrate the safety of primed cells in dogs, they can be considered for use in individual cases in the clinic. The lack of direct quantification of cytokines and other proteins contained in the mouse-derived tissue samples used for priming or secreted by the primed cADSCs is a major limitation of this study, but given the diversity of factors contained in the samples and the lack of clarity on the mechanism of action of cADSCs, an exhaustive proteomic analysis is needed to investigate them. Detailed analysis of the mechanisms by which inflammatory tissue enhances MSC function was not within the scope of this study, but should be conducted in the future. If the factors involved in MSC activation are identified for this priming method, they may be purified and combined, making this a disease-specific and scalable method. In addition, it will be necessary to investigate the effects of primed cADSCs on the function as well as phenotype of macrophages and Th cell subsets. However, to the best of our knowledge, this is the first study to report that exposure of cADSCs to colitis tissue appropriately enhances their immunomodulatory and therapeutic effects on the original disease, colitis. Based on this finding, better therapeutic efficacy may be achieved by priming MSCs with mucosal samples obtained in a relatively non-invasive manner by endoscopic gastrointestinal mucosal biopsy, an essential step in the definitive diagnosis of canine CIE and human IBD. To achieve this, further studies are needed to elucidate the mechanisms mediating the functional enhancement of MSCs by priming, as well as to analyze characteristics and functions of primed MSCs in more detail.

## 4 Materials and methods

### 4.1 cADSC isolation and characterization

cADSCs were isolated from the falciform ligament fat of five healthy beagles (males; mean age: 1.8 years; mean weight: 11.0 kg), as previously described (53). This study was approved by the Bioethics Committee of Nippon Veterinary and Life Science University (approval number 2023S-5; 31 March, 2023). The animals were handled in accordance with the animal care guidelines of the Institute of Laboratory Animal Resources, Nippon Veterinary and Life Science University, Japan. Briefly, collected adipose tissue was digested in Dulbecco's Modified Eagle's Medium (DMEM) containing 0.15% collagenase type I (Sigma-Aldrich, St. Louis, MO, USA) with gentle agitation at 37°C for 1 h. After centrifugation, the pellet containing the stromal vascular fraction was resuspended in DMEM, filtered through a 100 μm nylon mesh, plated into a T-75 culture flask containing DMEM complete medium [DMEM supplemented with 10% fetal bovine serum (FBS; Capricorn, Hessen, Germany) and 1% penicillin–streptomycin (Thermo Fisher Scientific, Waltham, MA, USA)], and incubated overnight in a humidified atmosphere with 5% CO_2_ at 37°C. The cells were detached using trypsin-EDTA solution (Sigma-Aldrich) upon reaching 80–90% confluence and passaged. As shown in our previous studies, cADSCs were characterized by flow cytometry for expression pattern of CD29, CD44, and CD90 positive, CD34, CD45, and HLA-DR negative cell surface markers (53). In addition, the trilineage differentiation potential of these cells for adipogenesis, osteogenesis, and chondrogenesis was confirmed. All experiments were performed using cADSCs at passages 2–3.

### 4.2 Mouse colon, liver homogenate, and serum preparation

To obtain mouse-derived samples and prepare homogenates for priming cADSCs, fifteen 6-week-old male C57BL/6J mice were purchased from Jackson Laboratory Japan (Kanagawa, Japan) and fed *ad libitum* on sterile water and standard experimental chow in a temperature- and light-controlled room. Mice in which colitis had been induced by 7 days of 3% DSS (36–50 kDa; MP Biomedical, Solon, OH, USA) solution administration were euthanized by CO_2_ asphyxiation, and colon, liver, and whole-blood samples were collected. The colon and liver samples were washed well in PBS and immediately frozen in liquid nitrogen, followed by manual homogenization on ice using a tapered tissue grinder. After adding 1 mL of PBS per 100 mg of homogenate, the samples were centrifuged at 10,000 × g for 20 min at 4°C, and the supernatant was collected and filtered through a 20 μm mesh filter for sterilization. The collected whole blood was centrifuged at 1,200 × g for 15 min at 4°C, and the supernatant was collected to obtain serum. Total protein concentrations of colon and liver homogenates and serum were determined using a Qubit protein assay kit (Thermo Fisher Scientific), in accordance with the manufacturer's instructions. Homogenates and sera were stored frozen in liquid nitrogen until use.

### 4.3 cADSC priming protocols

cADSCs were thawed and suspended in DMEM complete medium and plated on 100 mm cell culture dishes. After 3–5 days, when cADSCs reached 70–80% confluence, the medium was removed and washed twice with PBS, after which DMEM complete medium containing 0.1 mg of homogenate or serum as total protein per mL, or containing 20 ng of recombinant mouse TNF-α (Miltenyi Biotec, Bergisch Gladbach, Germany) and recombinant mouse IFN-γ (Miltenyi Biotec) per mL was added and incubated with 5% CO_2_ at 37°C. After 24 h, cells were detached with trypsin-EDTA solution and used for subsequent experiments.

### 4.4 Lymphocyte proliferation inhibition assay

cADSCs primed with colon homogenate at graded concentrations of 0.01 to 1.0 mg/mL, primed with liver homogenate or serum at concentrations of 0.1 mg/mL and 0.5 mg/mL, or primed with 20 ng/mL mouse TNF-α and mouse IFN-γ were plated in six-well plates. After 24 h, cADSC adhesion was confirmed and the medium of the wells was removed, followed by the addition of 1 × 10^6^ splenic Th cells from colitis mice without cADSC treatment pre-labeled with 5 μM Cell Trace Violet (Thermo Fisher Scientific) in RPMI-1640 complete medium (RPMI-1640 supplemented with 10% FBS, 1% penicillin–streptomycin, and 50 μM 2-mercaptoethanol) to the wells with or without 2 × 10^5^ naive or primed cADSCs. The cells were then co-cultured with or without cADSCs in the presence of anti-mouse CD3/CD28 antibody-loaded Anti-Biotin MACSiBead particles (Miltenyi Biotec) at 37°C with 5% CO_2_ for 72 h. Th cells and cADSCs were cultured with direct contact in the wells. Finally, Th cells were collected and stained with an anti-CD4-APC antibody (Clone: RM4-5; BioLegend) or the isotype control, and proliferation was measured by flow cytometry.

### 4.5 cADSC apoptosis assay

Naive cADSCs or cADSCs primed for 24 h with 20 ng/mL of each of TNF-α and IFN-γ, 0.1 mg/mL liver homogenate, or 0.1 mg/mL or 0.5 mg/mL colon homogenate were stained with the FITC Annexin-V Apoptosis Detection Kit with PI (BioLegend, San Diego, CA, USA), in accordance with the manufacturer's instructions, and then apoptotic cells were measured by flow cytometry as the sum of the percentages of cells positive for Annexin V alone or in combination with PI.

### 4.6 cADSC proliferation assay

Naive cADSCs or cADSCs primed for 24 h with 20 ng/mL of each of TNF-α and IFN-γ, 0.1 mg/mL liver homogenate, or 0.1 mg/mL or 0.5 mg/mL colon homogenate were plated in 96-well plates at 2 × 10^3^ cells each. After incubation at 37°C and 5% CO_2_ for 1–4 days, Cell Counting Kit-8 (CCK-8; Dojindo, Kumamoto, Japan) was added to the wells, in accordance with the manufacturer's instructions and cell proliferation was measured as OD values at a wavelength of 450 nm with a Synergy HT Microplate Reader (BioTek, Winooski, VT, USA). OD values after day 2 were normalized by OD values on day 1.

### 4.7 RNA sequencing

Total RNA was extracted from naive cADSCs or cADSCs primed for 24 h with 20 ng/mL of each of TNF-α and IFN-γ, or 0.1 mg/mL colon homogenate using a NucleoSpin RNA kit (TaKaRa, Shiga, Japan), in accordance with the manufacturer's instructions. Construction of the cDNA library was performed with 1 μg of total RNA using Illumina NEBNext Ultra II RNA Library Prep Kit, in accordance with the manufacturer's instructions, followed by paired-end sequencing (2 × 150 bp) using a NovaSeq 6000 instrument. An average of 16–20 million read pairs were generated for each library. Primary analysis of RNA-seq raw data was carried out on the web using RaNa-seq (https://ranaseq.eu/; accessed on 22 February, 2024) (54). In this way, FASTQ files were pre-processed with the FASTp tool (55), expression was quantified with Salmon (56) against the genome assembly CanFam3.1, and raw count data and count data normalized by transcripts per million (TPM) were output. Secondary analysis of RNA-seq data was performed on the web by inputting these count data into RNAseqChef (https://imeg-ku.shinyapps.io/RNAseqChef/; accessed on 10 March, 2024) (57). For the pairwise comparison analysis, DEG detection was performed using DESeq2 with fold change and false discovery rate (FDR) cut-off values of >2 and < 0.05, respectively. To compare DEGs from different data sets, Venn diagram analysis was performed. Functional enrichment analysis was performed using clusterProfiler (58) with an FDR cut-off of >0.05 based on the MSigDB hallmark gene set.

### 4.8 Mouse colitis induction and cADSC treatment

To evaluate the therapeutic effect of cADSCs primed with each condition on colitis *in vivo*, 35 6-week-old c57BL/6J mice were purchased separately from the mice used for homogenate preparation and lymphocyte proliferation inhibition assays. To induce colitis, mice received 3% DSS solution in drinking water for 7 days, followed by sterile water for 3 days. On day 2 of colitis induction, mice received 200 μL of PBS or 2 × 10^6^ cells of naive or primed cADSCs intraperitoneally. In contrast, healthy control mice received only sterile water to drink and the intraperitoneal administration of PBS. Each group included 5 mice. The mice were sacrificed by CO_2_ asphyxiation on day 10, and colon tissues and immune cells were harvested for subsequent histological evaluation and phenotypic analysis.

### 4.9 Evaluation of colitis severity

The severity of colitis was assessed based on the DAI, colon length, and the histological score of the colon. The DAI was calculated as the sum of three scores, namely, percent weight loss from initial weight (grades 0–4: 0, none; 1, 1–5% loss; 2, 6–10% loss; 3, 11–20% loss; 4, >20% loss), stool consistency (grades 0–3: 0, normal; 1, soft; 2, loose; 3, watery), and rectal bleeding (grades 0–3: 0, none; 1, occult blood; 2, visible bleeding; 3, gross bleeding), and was compared among the groups. Colon length was measured with a ruler from colon samples harvested on day 10. Two pathologists evaluated hematoxylin and eosin-stained colon tissue sections in a blinded fashion, and histological scores were calculated in accordance with previously reported methods (23) as follows: inflammatory cell infiltration (score 0–4: 0, no filtration; 1, infiltrate around crypt bases; 2, infiltrate reaching the lamina muscularis mucosa with abundant edema; 3, extensive infiltration reaching the muscularis mucosa with abundant edema; 4, infiltration of the submucosal layer) and epithelial damage (score 0–4: 0, normal morphology; 1, loss of goblet cells; 2, loss of goblet cells in large areas; 3, loss of crypts; 4, loss of crypts in large areas).

### 4.10 Phenotypic analysis of mouse peritoneal macrophages and splenic CD4^+^ Th cells

To investigate the effect of primed cADSCs on the immune system of mice with DSS-induced colitis, immune cells were harvested from these mice with and without cADSC treatment. Isolation and phenotypic analysis of mouse peritoneal macrophages and splenic CD4^+^ Th cells were performed as previously described (24). In brief, peritoneal macrophages were isolated by collecting and centrifuging PBS injected into the abdominal cavity of mice, and splenic CD4^+^ Th cells were isolated from cell suspensions obtained by mechanical mashing of the spleen by negative magnetic selection using a mouse CD4^+^ T-cell isolation kit (Miltenyi Biotec), in accordance with the manufacturer's instructions.

For flow cytometric phenotyping of macrophages, the cells were stained with the following antibodies or their respective isotype controls: anti-F4/80-FITC (clone: BM8; BioLegend), anti-CD80-APC (clone: 16-10A1; BioLegend), and anti-CD206-PE (clone: C068C2; BioLegend).

For intracellular cytokine detection, Th cells were stimulated with phorbol 12-myristate 13-acetate (50 ng/mL; Sigma-Aldrich) and ionomycin (1 μg/mL; Sigma-Aldrich) for 6 h and brefeldin A (10 μg/mL; Sigma-Aldrich) for 4 h, and then stained with an anti-CD4-APC antibody (Clone: RM4-5; BioLegend) or isotype control. The cells were fixed and permeabilized with Cyto-Fast Fix/Perm Buffer Set (BioLegend) and then stained with anti-IFN-γ-PE (clone: XMG1.2; BioLegend), anti-IL-4-PE (clone: 11B11; BioLegend), and anti-IL-17A-PE (clone: TC11-18H10.1; BioLegend) antibodies, or their respective isotype controls.

For flow cytometric detection of Treg cells, Th cells were stained with anti-CD4-APC and anti-CD25-PE (clone: PC61; BioLegend) antibodies or their respective isotype controls, followed by fixing and permeabilizing with True-Nuclear Transcription Factor Buffer Set (BioLegend) and staining with an anti-Foxp3-Alexa Fluor488 antibody (clone: MF-14; BioLegend) or isotype control. All samples were measured using a CytoFLEX instrument (Beckman Coulter, Brea, CA, USA) and data were analyzed using CytExpert ver. 2.0 analysis software.

### 4.11 Statistical analysis

All results are presented as the mean ± standard deviation. Data were tested for distribution normality using the Shapiro-Wilks normality test. Differences among multiple groups were assessed by one-way analysis of variance for normally distributed data and compared using the Dunnett's or Tukey–Kramer multiple comparison *post hoc* test, and assessed by Kruskal-Wallis test for non-normally distributed data and compared using Steel-Dwass post hock test. *p* < 0.05 was considered statistically significant. Statistical analyses were performed using R Commander 4.1.2.

## Data availability statement

The original contributions presented in the study are included in the article/Supplementary material, further inquiries can be directed to the corresponding author.

## Ethics statement

The animal study was approved by the Bioethics Committee of Nippon Veterinary and Life Science University. The study was conducted in accordance with the local legislation and institutional requirements.

## Author contributions

YY: Conceptualization, Data curation, Formal analysis, Investigation, Methodology, Project administration, Validation, Visualization, Writing – original draft. TT: Conceptualization, Data curation, Formal analysis, Funding acquisition, Investigation, Methodology, Project administration, Resources, Supervision, Visualization, Writing – review & editing. TN: Investigation, Writing – review & editing. MM: Investigation, Writing – review & editing. YT: Investigation, Writing – review & editing. RS: Investigation, Writing – review & editing. HM: Investigation, Writing – review & editing.

## References

[1] Chang JT. Pathophysiology of inflammatory bowel diseases. N Engl J Med. (2020) 383:2652–64. 10.1056/NEJMra2002697 33382932

[2] Catalan-Serra I Brenna Ø. Immunotherapy in inflammatory bowel disease: novel and emerging treatments. Hum Vaccin Immunother. (2018) 14:2597–611. 10.1080/21645515.2018.1461297 29624476 PMC6314405

[3] Jergens AE Heilmann RM. Canine chronic enteropathy-current state-of-the-art and emerging concepts. Front Vet Sci. (2022) 9:923013. 10.3389/fvets.2022.923013 36213409 PMC9534534

[4] Dandrieux JRS Mansfield CS. Chronic enteropathy in canines: prevalence, impact and management strategies. Vet Med (Auckl). (2019) 10:203–14. 10.2147/VMRR.S162774 31828025 PMC6902862

[5] Mastrolia I Foppiani EM Murgia A Candini O Samarelli AV Grisendi G. Challenges in clinical development of mesenchymal stromal/stem cells: concise review. Stem Cells Transl Med. (2019) 8:1135–48. 10.1002/sctm.19-0044 31313507 PMC6811694

[6] Voga M Adamic N Vengust M Majdic G. Stem cells in veterinary medicine-current state and treatment options. Front Vet Sci. (2020) 7:278. 10.3389/fvets.2020.00278 32656249 PMC7326035

[7] Bunnell BA. Adipose tissue-derived mesenchymal stem cells. Cells. (2021) 10:3433. 10.3390/cells10123433 34943941 PMC8700397

[8] Lopez-Santalla M Garin MI. Improving the efficacy of mesenchymal stem/stromal-based therapy for treatment of inflammatory bowel diseases. Biomedicines. (2021) 9:1507. 10.3390/biomedicines9111507 34829736 PMC8615066

[9] Ciccocioppo R Baumgart DC Dos Santos CC Galipeau J Klersy C Orlando G. Perspectives of the international society for cell & gene therapy gastrointestinal scientific committee on the intravenous use of mesenchymal stromal cells in inflammatory bowel disease (PeMeGi). Cytotherapy. (2019) 21:824–39. 10.1016/j.jcyt.2019.05.003 31201092

[10] Teshima T. Heterogeneity of mesenchymal stem cells as a limiting factor in their clinical application to inflammatory bowel disease in dogs and cats. Vet J. (2024) 304:106090. 10.1016/j.tvjl.2024.106090 38417670

[11] Zhou T Yuan Z Weng J Pei D Du X He C. Challenges and advances in clinical applications of mesenchymal stromal cells. J Hematol Oncol. (2021) 14:24. 10.1186/s13045-021-01037-x 33579329 PMC7880217

[12] Pérez-Merino EM Usón-Casaús JM Zaragoza-Bayle C Duque-Carrasco J Mariñas-Pardo L Hermida-Prieto M. Safety and efficacy of allogeneic adipose tissue-derived mesenchymal stem cells for treatment of dogs with inflammatory bowel disease: Clinical and laboratory outcomes. Vet J. (2015) 206:385–90. 10.1016/j.tvjl.2015.08.003 26526522

[13] Cristóbal JI Duque FJ Usón-Casaús JM Ruiz P Nieto EL Pérez-Merino EM. Effects of allogeneic mesenchymal stem cell transplantation in dogs with inflammatory bowel disease treated with and without corticosteroids. Animals (Basel). (2021) 11:2061. 10.3390/ani11072061 34359189 PMC8300310

[14] Noronha NC Mizukami A Caliári-Oliveira C Cominal JG Rocha JLM Covas DT. Priming approaches to improve the efficacy of mesenchymal stromal cell-based therapies. Stem Cell Res Ther. (2019) 10:131. 10.1186/s13287-019-1224-y 31046833 PMC6498654

[15] López-García L Castro-Manrreza ME. TNF-α and IFN-γ participate in improving the immunoregulatory capacity of mesenchymal stem/stromal cells: importance of cell-cell contact and extracellular vesicles. Int J Mol Sci. (2021) 22:9531. 10.3390/ijms22179531 34502453 PMC8431422

[16] Klimczak A Kozlowska U. Mesenchymal stromal cells and tissue-specific progenitor cells: their role in tissue homeostasis. Stem Cells Int. (2016) 2016:4285215. 10.1155/2016/4285215 26823669 PMC4707334

[17] Shi Y Su J Roberts AI Shou P Rabson AB Ren G. How mesenchymal stem cells interact with tissue immune responses. Trends Immunol. (2012) 33:136–43. 10.1016/j.it.2011.11.004 22227317 PMC3412175

[18] Bernardo ME Fibbe WE. Mesenchymal stromal cells: sensors and switchers of inflammation. Cell Stem Cell. (2013) 13:392–402. 10.1016/j.stem.2013.09.006 24094322

[19] Lin Y Yang X Yue W Xu X Li B Zou L. Chemerin aggravates DSS-induced colitis by suppressing M2 macrophage polarization. Cell Mol Immunol. (2014) 11:355–66. 10.1038/cmi.2014.15 24727542 PMC4085517

[20] Yang F Wang D Li Y Sang L Zhu J Wang J. Th1/Th2 balance and Th17/Treg-mediated immunity in relation to murine resistance to dextran sulfate-induced colitis. J Immunol Res. (2017) 2017:7047201. 10.1155/2017/7047201 28584821 PMC5444015

[21] Yang HM Song WJ Li Q Kim SY Kim HJ Ryu MO. Canine mesenchymal stem cells treated with TNF-α and IFN-γ enhance anti-inflammatory effects through the COX-2/PGE2 pathway. Res Vet Sci. (2018) 119:19–26. 10.1016/j.rvsc.2018.05.011 29783120

[22] Song WJ Li Q Ryu MO Nam A An JH Jung YC. Canine adipose tissue-derived mesenchymal stem cells pre-treated with TNF-alpha enhance immunomodulatory effects in inflammatory bowel disease in mice. Res Vet Sci. (2019) 125:176–84. 10.1016/j.rvsc.2019.06.012 31247473 PMC7111869

[23] An JH Li Q Bhang DH Song WJ Youn HY. TNF-α and INF-γ primed canine stem cell-derived extracellular vesicles alleviate experimental murine colitis. Sci Rep. (2020) 10:2115. 10.1038/s41598-020-58909-4 32034203 PMC7005871

[24] Alex P Zachos NC Nguyen T Gonzales L Chen TE Conklin LS. Distinct cytokine patterns identified from multiplex profiles of murine DSS and TNBS-induced colitis. Inflamm Bowel Dis. (2009) 15:341–52. 10.1002/ibd.20753 18942757 PMC2643312

[25] Kim JJ Shajib MS Manocha MM Khan WI. Investigating intestinal inflammation in DSS-induced model of IBD. J Vis Exp. (2012) 3678. 10.3791/3678-v 22331082 PMC3369627

[26] Sayegh S El Atat O Diallo K Rauwel B Degboé Y Cavaignac E. Rheumatoid synovial fluids regulate the immunomodulatory potential of adipose-derived mesenchymal stem cells through a TNF/NF-κB-dependent mechanism. Front Immunol. (2019) 10:1482. 10.3389/fimmu.2019.01961 31316519 PMC6611153

[27] Silva-Carvalho AÉ Rodrigues LP Schiavinato JL Alborghetti MR Bettarello G Simões BP. GVHD-derived plasma as a priming strategy of mesenchymal stem cells. Stem Cell Res Ther. (2020) 11:156. 10.1186/s13287-020-01659-x 32299501 PMC7164240

[28] Li X Shang B Li YN Shi Y Shao C. IFNγ and TNFα synergistically induce apoptosis of mesenchymal stem/stromal cells via the induction of nitric oxide. Stem Cell Res Ther. (2019) 10:18. 10.1186/s13287-018-1102-z 30635041 PMC6330503

[29] Domenis R Cifù A Quaglia S Pistis C Moretti M Vicario A. Pro inflammatory stimuli enhance the immunosuppressive functions of adipose mesenchymal stem cells-derived exosomes. Sci Rep. (2018) 8:13325. 10.1038/s41598-018-31707-9 30190615 PMC6127134

[30] Montesinos JJ López-García L Cortés-Morales VA Arriaga-Pizano L Valle-Ríos R Fajardo-Orduña GR. Human bone marrow mesenchymal stem/stromal cells exposed to an inflammatory environment increase the expression of ICAM-1 and release microvesicles enriched in this adhesive molecule: analysis of the participation of TNF-α and IFN-γ. J Immunol Res. (2020) 2020:8839625. 10.1155/2020/8839625 33335929 PMC7723491

[31] Prasanna SJ Gopalakrishnan D Shankar SR Vasandan AB. Pro-inflammatory cytokines, IFNgamma and TNFalpha, influence immune properties of human bone marrow and Wharton jelly mesenchymal stem cells differentially. PLoS ONE. (2010) 5:e9016. 10.1371/journal.pone.0009016 20126406 PMC2814860

[32] Takeshita K Motoike S Kajiya M Komatsu N Takewaki M Ouhara K. Xenotransplantation of interferon-gamma-pretreated clumps of a human mesenchymal stem cell/extracellular matrix complex induces mouse calvarial bone regeneration. Stem Cell Res Ther. (2017) 8:101. 10.1186/s13287-017-0550-1 28446226 PMC5406942

[33] Lavrentieva A Majore I Kasper C Hass R. Effects of hypoxic culture conditions on umbilical cord-derived human mesenchymal stem cells. Cell Commun Signal. (2010) 8:18. 10.1186/1478-811X-8-18 20637101 PMC2918620

[34] Li B Li C Zhu M Zhang Y Du J Xu Y. Hypoxia-induced mesenchymal stromal cells exhibit an enhanced therapeutic effect on radiation-induced lung injury in mice due to an increased proliferation potential and enhanced antioxidant ability. Cell Physiol Biochem. (2017) 44:1295–310. 10.1159/000485490 29183009

[35] Sivanathan KN Rojas-Canales D Grey ST Gronthos S Coates PT. Transcriptome profiling of IL-17A preactivated mesenchymal stem cells: a comparative study to unmodified and ifn-γ modified mesenchymal stem cells. Stem Cells Int. (2017) 2017:1025820. 10.1155/2017/1025820 28293262 PMC5331321

[36] Pourgholaminejad A Aghdami N Baharvand H Moazzeni SM. The effect of pro-inflammatory cytokines on immunophenotype, differentiation capacity and immunomodulatory functions of human mesenchymal stem cells. Cytokine. (2016) 85:51–60. 10.1016/j.cyto.2016.06.003 27288632

[37] Crop MJ Baan CC Korevaar SS Ijzermans JN Pescatori M Stubbs AP. Inflammatory conditions affect gene expression and function of human adipose tissue-derived mesenchymal stem cells. Clin Exp Immunol. (2010) 162:474–86. 10.1111/j.1365-2249.2010.04256.x 20846162 PMC3026550

[38] Okayasu I Hatakeyama S Yamada M Ohkusa T Inagaki Y Nakaya R. novel method in the induction of reliable experimental acute and chronic ulcerative colitis in mice. Gastroenterology. (1990) 98:694–702. 10.1016/0016-5085(90)90290-H 1688816

[39] Yasumura Y Teshima T Nagashima T Michishita M Takano T Taira Y. Immortalized canine adipose-derived mesenchymal stem cells maintain the immunomodulatory capacity of the original primary cells. Int J Mol Sci. (2023) 24:17484. 10.3390/ijms242417484 38139314 PMC10743981

[40] Randhawa PK Singh K Singh N Jaggi AS. A review on chemical-induced inflammatory bowel disease models in rodents. Korean J Physiol Pharmacol. (2014) 18:279–88. 10.4196/kjpp.2014.18.4.279 25177159 PMC4146629

[41] Katsandegwaza B Horsnell W Smith K. Inflammatory bowel disease: a review of pre-clinical murine models of human disease. Int J Mol Sci. (2022) 23:9344. 10.3390/ijms23169344 36012618 PMC9409205

[42] Dieleman LA Ridwan BU Tennyson GS Beagley KW Bucy RP Elson CO. Dextran sulfate sodium-induced colitis occurs in severe combined immunodeficient mice. Gastroenterology. (1994) 107:1643–52. 10.1016/0016-5085(94)90803-6 7958674

[43] Yabana T Arimura Y Tanaka H Goto A Hosokawa M Nagaishi K. Enhancing epithelial engraftment of rat mesenchymal stem cells restores epithelial barrier integrity. J Pathol. (2009) 218:350–9. 10.1002/path.2535 19291714

[44] Krampera M. Mesenchymal stromal cell 'licensing': a multistep process. Leukemia. (2011) 25:1408–14. 10.1038/leu.2011.108 21617697

[45] Castelo-Branco MT Soares ID Lopes DV Buongusto F Martinusso CA do Rosario A Jr. Intraperitoneal but not intravenous cryopreserved mesenchymal stromal cells home to the inflamed colon and ameliorate experimental colitis. PLoS ONE. (2012) 7:e33360. 10.1371/journal.pone.0033360 22432015 PMC3303821

[46] Li Q Lian Y Deng Y Chen J Wu T Lai X. mRNA-engineered mesenchymal stromal cells expressing CXCR2 enhances cell migration and improves recovery in IBD. Mol Ther Nucleic Acids. (2021) 26:222–36. 10.1016/j.omtn.2021.07.009 34513306 PMC8413681

[47] Gonçalves Fda C Schneider N Pinto FO Meyer FS Visioli F Pfaffenseller B. Intravenous vs intraperitoneal mesenchymal stem cells administration: what is the best route for treating experimental colitis? World J Gastroenterol. (2014) 20:18228–39. 10.3748/wjg.v20.i48.18228 25561790 PMC4277960

[48] Pan XH Li QQ Zhu XQ Li ZA Cai XM Pang RQ. Mechanism and therapeutic effect of umbilical cord mesenchymal stem cells in inflammatory bowel disease. Sci Rep. (2019) 9:17646. 10.1038/s41598-019-54194-y 31776475 PMC6881332

[49] Sanchez-Diaz M Quiñones-Vico MI Sanabria de la Torre R Montero-Vílchez T Sierra-Sánchez A Molina-Leyva A. Biodistribution of mesenchymal stromal cells after administration in animal models and humans: a systematic review. J Clin Med. (2021) 10:2925. 10.3390/jcm10132925 34210026 PMC8268414

[50] Siel D Beltrán CJ Martínez E Pino M Vargas N Salinas A. Elucidating the role of innate and adaptive immune responses in the pathogenesis of canine chronic inflammatory enteropathy-a search for potential biomarkers. Animals (Basel). (2022) 12:1645. 10.3390/ani12131645 35804545 PMC9264988

[51] Uko V Thangada S Radhakrishnan K. Liver disorders in inflammatory bowel disease. Gastroenterol Res Pract. (2012) 2012:642923. 10.1155/2012/642923 22474447 PMC3296398

[52] Gäbele E Dostert K Hofmann C Wiest R Schölmerich J Hellerbrand C. DSS induced colitis increases portal LPS levels and enhances hepatic inflammation and fibrogenesis in experimental NASH. J Hepatol. (2011) 55:1391–9. 10.1016/j.jhep.2011.02.035 21703208

[53] Yasumura Y Teshima T Taira Y Saito T Yuchi Y Suzuki R. Optimal intravenous administration procedure for efficient delivery of canine adipose-derived mesenchymal stem cells. Int J Mol Sci. (2022) 23:14681. 10.3390/ijms232314681 36499004 PMC9740176

[54] Prieto C Barrios D. RaNA-Seq: interactive RNA-Seq analysis from FASTQ files to functional analysis. Bioinformatics. (2019) 36:1955–1956. 10.1093/bioinformatics/btz854 31730197

[55] Chen S Zhou Y Chen Y Gu J. fastp: an ultra-fast all-in-one FASTQ preprocessor. Bioinformatics. (2018) 34:i884–90. 10.1093/bioinformatics/bty560 30423086 PMC6129281

[56] Patro R Duggal G Love MI Irizarry RA Kingsford C. Salmon provides fast and bias-aware quantification of transcript expression. Nat Methods. (2017) 14:417–9. 10.1038/nmeth.4197 28263959 PMC5600148

[57] Etoh K Nakao M A. web-based integrative transcriptome analysis, RNAseqChef, uncovers the cell/tissue type-dependent action of sulforaphane. J Biol Chem. (2023) 299:104810. 10.1016/j.jbc.2023.104810 37172729 PMC10267603

[58] Wu T Hu E Xu S Chen M Guo P Dai Z. clusterProfiler 40: A universal enrichment tool for interpreting omics data. Innovation (Camb). (2021) 2:100141. 10.1016/j.xinn.2021.100141 34557778 PMC8454663

