# Supplementary material for: Effective enhancement of the immunomodulatory capacity of canine adipose-derived mesenchymal stromal cells on colitis by priming with colon tissue from mice with colitis
Source: Front Vet Sci. 2024 Aug 8;11:1437648. doi: 10.3389/fvets.2024.1437648 (PMC11338805; doi:10.3389/fvets.2024.1437648)
Supplement: Supplementary file 1 [file Data_Sheet_1.docx]

Supplementary Material


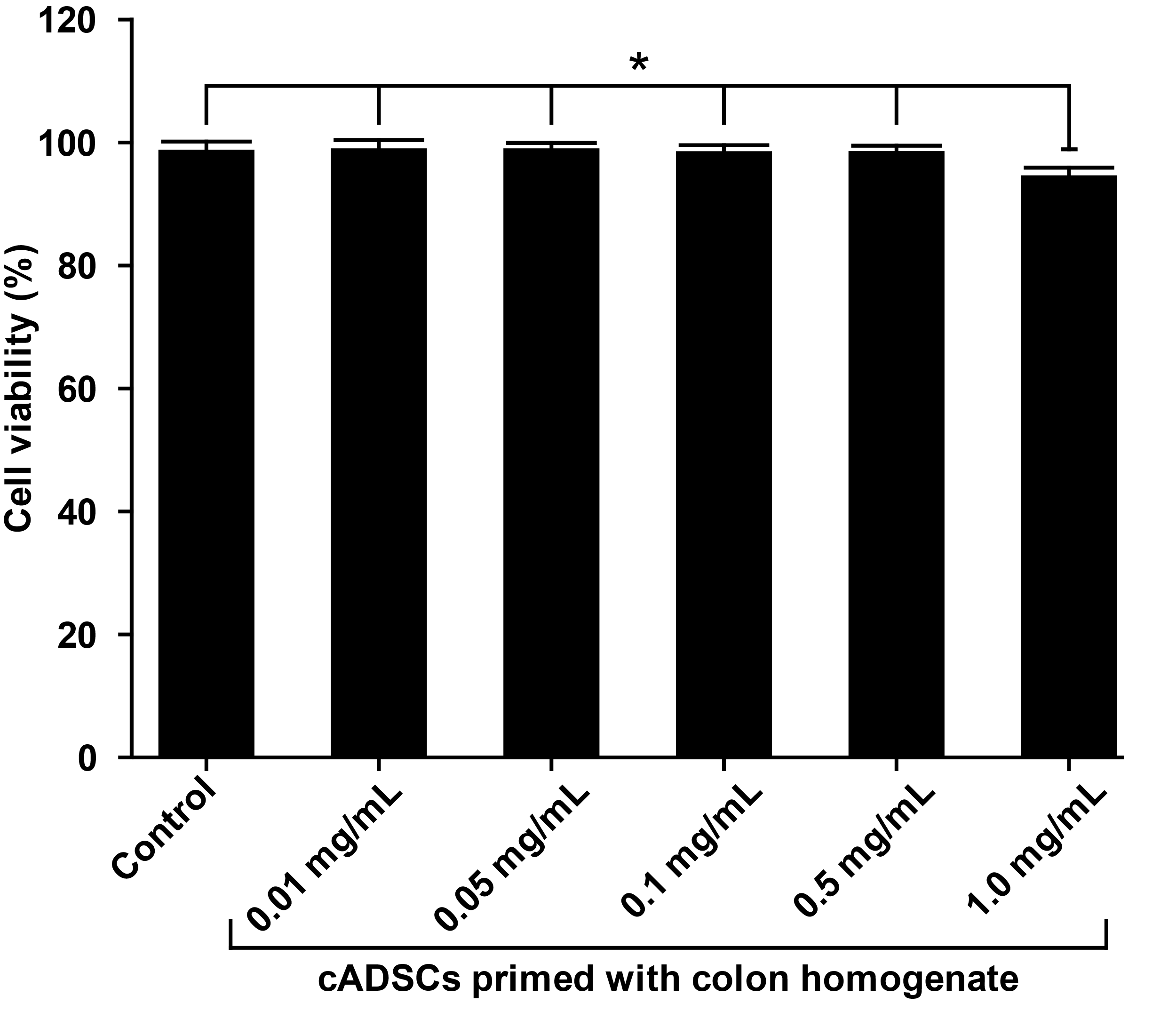


**Supplementary Figure 1.** Cell viability of cADSCs primed with colon homogenates at graded concentrations for 24 h. Cell viability was calculated as (number of viable cells) / (number of viable cells + number of dead cells) as measured by the trypan blue exclusion method. Data are expressed as the mean ± standard deviation from experiments performed in triplicate; *n* = 5; **p* < 0.05.


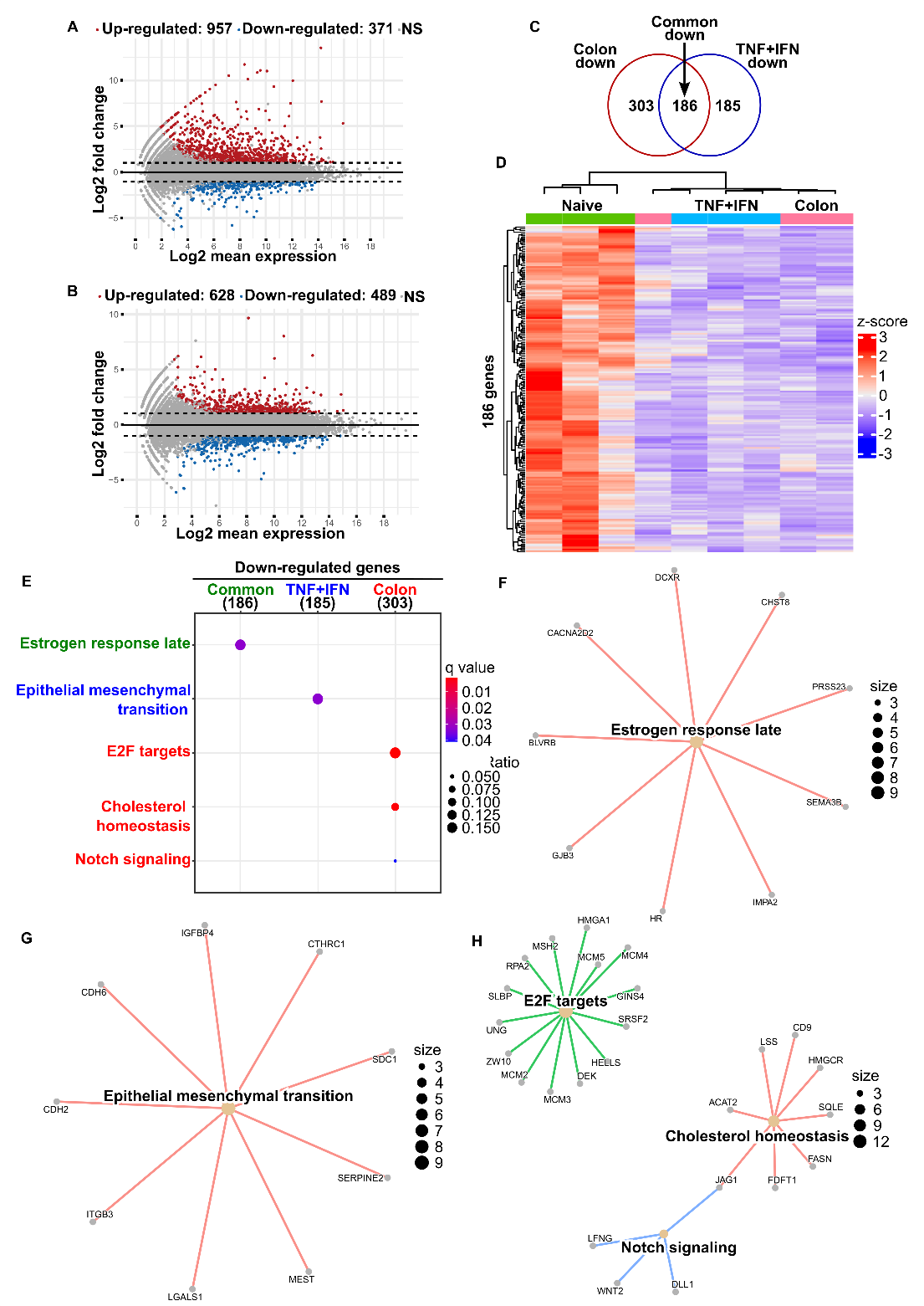


**Supplementary Figure 2**. Effect of different priming conditions on the transcriptome of cADSCs. **(A)** MA plot from RNA-sequencing (RNA-seq) data of naive and TNF-α+IFN-γ primed cADSCs; FDR < 0.05. **(B)** MA plot from RNA-seq data of naive and colitis-colon primed cADSCs; FDR < 0.05. **(C)** Venn diagram of genes significantly downregulated by TNF-α+IFN-γ or colon priming. **(D)** Integrated heatmap of genes commonly downregulated in both priming, **(E)** Functional pathways enriched in gene sets downregulated by priming, including 186 common genes (green), 185 TNF-α+IFN-γ priming specific genes (blue), and 303 colon priming specific genes (red); FDR < 0.05. **(F)** cnet plot of pathways enriched for downregulated gene sets common to each priming. **(G)** cnet plot of pathways enriched for TNF-α+IFN-γ priming-specific downregulated gene sets. **(H)** cnet plot of pathways enriched for colon priming-specific downregulated gene sets.


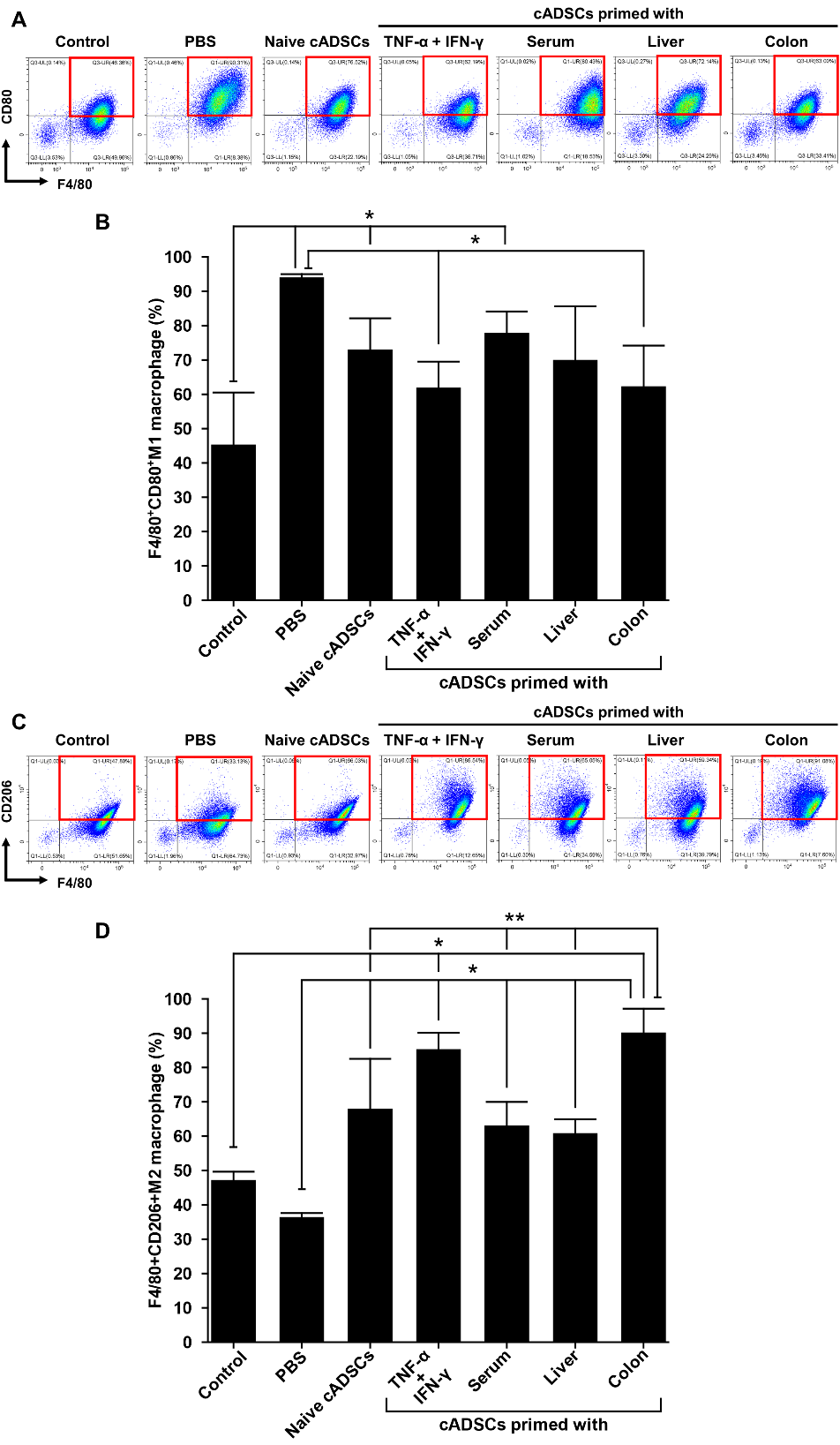


**Supplementary Figure 3.** Differential capacity of cADSCs primed with variable conditions to modulate macrophage polarity. **(A)** Flow cytometric determination of the frequency of F4/80^+^CD80^+^ M1 macrophages. **(B)** cADSCs primed with TNF-α+IFN-γ or colon homogenate significantly suppressed macrophage M1 polarization. **(C)** F4/80^+^CD206^+^ M2 macrophage frequency determined by flow cytometry. **(D)** M2 macrophages were significantly induced in all cADSC treatment groups. Data are expressed as the mean ± standard deviation from experiments performed in triplicate; *n* = 5; **p* < 0.05, ***p* < 0.01.


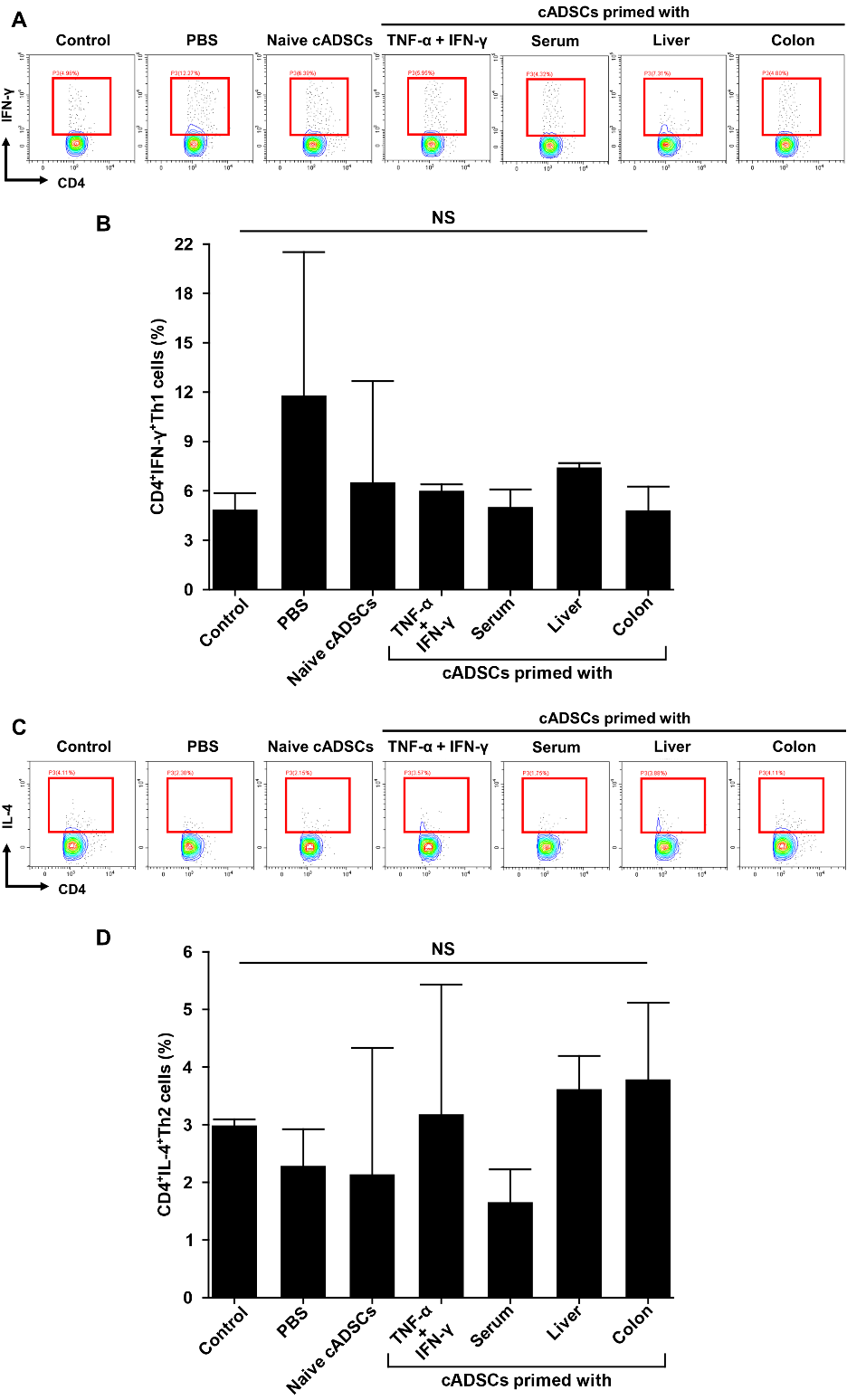


**Supplementary Figure 4.** Differential capacity of cADSCs primed with various conditions to modulate Th1/Th2-cell balance. (**A**) Flow cytometric determination of the frequency of CD4^+^ IFN-γ^+^ Th1 cells in mice treated with cADSCs primed under different conditions. **(B)** All of the cADSC treatments reduced Th1 cells in the mice, but the difference was not statistically significant. **(C)** Flow cytometric determination of the frequency of CD4^+^ IL-4^+^ Th2 cells **(D)** No apparent effect on Th2-cell frequency was found with the administration of primed cADSCs. Data are expressed as the mean ± standard deviation from experiments performed in triplicate; *n* = 5; N.S. = not significant.


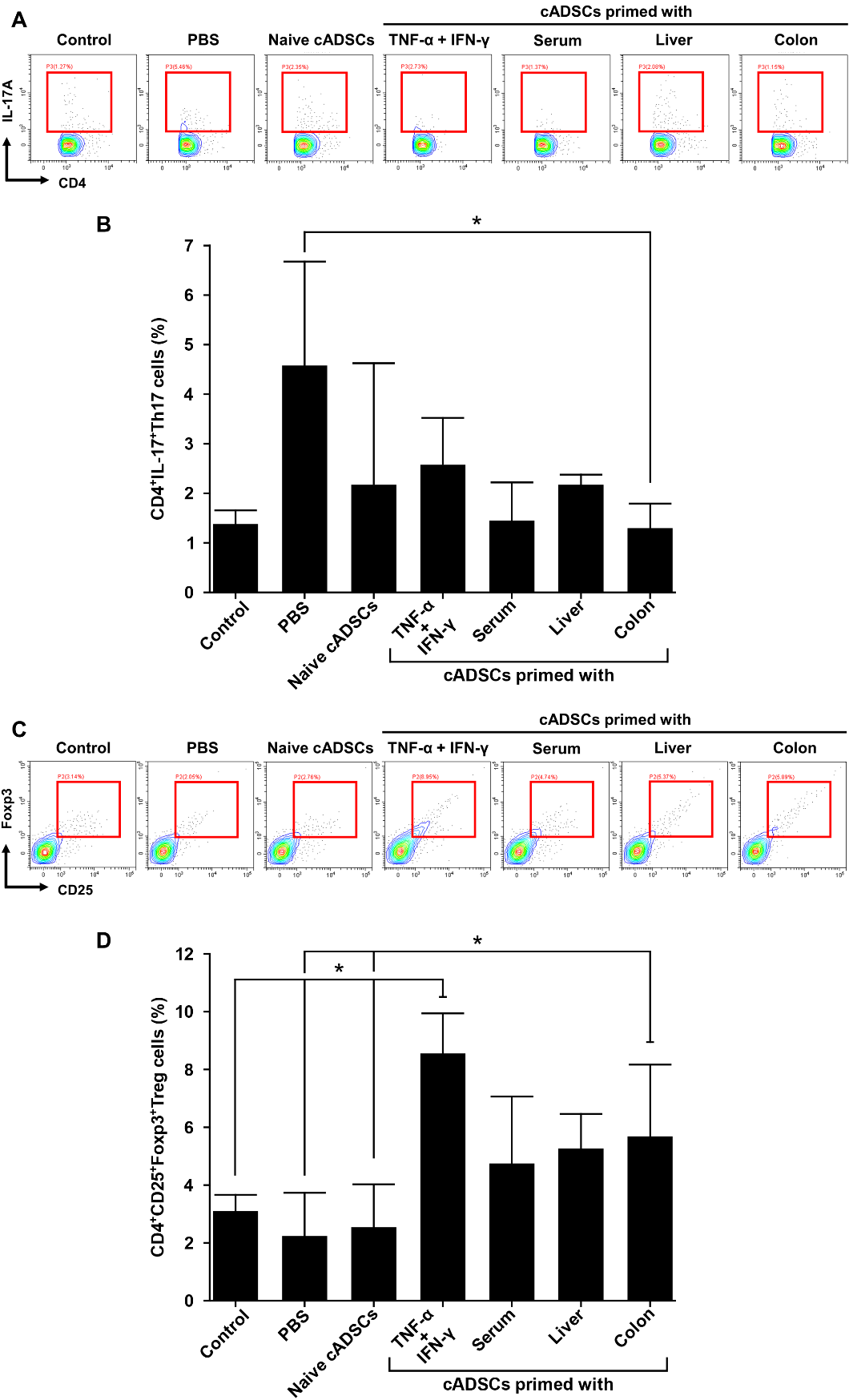


**Supplementary Figure 5.** Effect of primed cADSCs on the Th17/Treg-cell paradigm in mice with colitis. **(A)** Flow cytometric determination of the frequency of CD4^+^IL-17A^+^ Th17 cells. **(B)** The frequency of Th17 cells was significantly reduced by treatment with colon-primed cADSCs. **(C)** Treg cells were detected by flow cytometry as CD4-positive cells co-expressing CD25 and Foxp3. **(D)** cADSCs primed with TNF-α+IFN-γ or colon homogenate greatly induced Treg cells in colitis mice. Data are expressed as the mean ± standard deviation from experiments performed in triplicate; *n* = 5; **p* < 0.05.
